# Supplementary material for: Household coverage, quality and costs of care provided by community health worker teams and the determining factors: findings from a mixed methods study in South Africa
Source: BMJ Open. 2020 Aug 20;10(8):e035578. doi: 10.1136/bmjopen-2019-035578 (PMC7440700; doi:10.1136/bmjopen-2019-035578)
Supplement: Author's manuscript [file bmjopen-2019-035578.draft_revisions.pdf]

BMJ Open is committed to open peer review. As part of this commitment we make the peer review history of every article we publish publicly available.

When an article is published we post the peer reviewers' comments and the authors' responses online. We also post the versions of the paper that were used during peer review. These are the versions that the peer review comments apply to.

The versions of the paper that follow are the versions that were submitted during the peer review process. They are not the versions of record or the final published versions. They should not be cited or distributed as the published version of this manuscript.

BMJ Open is an open access journal and the full, final, typeset and author-corrected version of record of the manuscript is available on our site with no access controls, subscription charges or pay-per-view fees (http://bmjopen.bmj.com).

If you have any questions on BMJ Open's open peer review process please email info.bmjopen@bmj.com

BMJ Open

Household coverage, quality and costs of care provided by CHW teams and the determining factors: Findings from a mixed methods study in South Africa

Journal:	*BMJ Open*
Manuscript ID	bmjopen-2019-035578
Article Type:	Original research
Date Submitted by the Author:	11-Nov-2019

Complete List of Authors:	Goudge, Jane; University of the Witwatersrand School of Public Health, Centre for Health Policy<br>Thorogood, Margaret; University of Warwick, Medical School<br>de Kadt, Julia; University of the Witwatersrand, Gauteng City-Region Observatory<br>Malatji, Hlologelo; University of the Witwatersrand School of Public Health, Centre for Health Policy; University of the Witwatersrand Centre for Health Policy,<br>Babalola, Olukemi; University of the Witwatersrand School of Public Health, Centre for Health Policy<br>Tseng, Yu-hwei; University of the Witwatersrand, Centre for Health Policy, School of Public Health, Faculty of Health Sciences<br>Watkins, Jocelyn ; University of Warwick,<br>Muteba, Michel; University of the Witwatersrand, School of Public Health, Faculty of Health Sciences<br>Rwafa, Teurai; University of the Witwatersrand, Centre for Health Policy, School of Public Health, Faculty of Health Sciences<br>Daviaud, Emmanuelle; South African Medical Research Council, Health Systems<br>Levin, Jonathan; University of the Witwatersrand, School of Public Health, Faculty of Health Sciences<br>Nxumalo, Nonhlanhla; University of the Witwatersrand, Centre for Health Policy; Centre for Health Policy<br>Griffiths, Frances; University of Warwick, Warwick Medical School

Keywords:	Organisation of health services < HEALTH SERVICES ADMINISTRATION & MANAGEMENT, Quality in health care < HEALTH SERVICES ADMINISTRATION & MANAGEMENT, Human resource management < HEALTH SERVICES ADMINISTRATION & MANAGEMENT

SCHOLARONE™
Manuscripts

I, the Submitting Author has the right to grant and does grant on behalf of all authors of the Work (as defined in the below author licence), an exclusive licence and/or a non-exclusive licence for contributions from authors who are: i) UK Crown employees; ii) where BMJ has agreed a CC-BY licence shall apply, and/or iii) in accordance with the terms applicable for US Federal Government officers or employees acting as part of their official duties; on a worldwide, perpetual, irrevocable, royalty-free basis to BMJ Publishing Group Ltd ("BMJ") its licensees and where the relevant Journal is co-owned by BMJ to the co-owners of the Journal, to publish the Work in this journal and any other BMJ products and to exploit all rights, as set out in our licence.

The Submitting Author accepts and understands that any supply made under these terms is made by BMJ to the Submitting Author unless you are acting as an employee on behalf of your employer or a postgraduate student of an affiliated institution which is paying any applicable article publishing charge ("APC") for Open Access articles. Where the Submitting Author wishes to make the Work available on an Open Access basis (and intends to pay the relevant APC), the terms of reuse of such Open Access shall be governed by a Creative Commons licence – details of these licences and which Creative Commons licence will apply to this Work are set out in our licence referred to above.

Other than as permitted in any relevant BMJ Author's Self Archiving Policies, I confirm this Work has not been accepted for publication elsewhere, is not being considered for publication elsewhere and does not duplicate material already published. I confirm all authors consent to publication of this Work and authorise the granting of this licence.

For peer review only

Title: Household coverage, quality and costs of care provided by CHW teams and the determining factors: Findings from a mixed methods study in South Africa

Authors: Jane Goudge[1], Julia de Kadt[1], Olukemi Babalola[1], Michel Muteba[2], Yu-hwei Tseng[1], Hlologelo Malatji[1], Teurai Rwafa[1], Nonhlanhla Nxumalo[1], Jonathan Levin[2], Margaret Thorogood[1,3], Emmanuelle Daviaud[4], Jocelyn Anstey Watkins[3], Frances Griffiths[1, 3].

Affiliations
1. Centre for Health Policy, School of Public Health, Faculty of Health Sciences, University of the Witwatersrand, Johannesburg
2. School of Public Health, Faculty of Health Sciences, University of the Witwatersrand, Johannesburg
3. Warwick Medical School, Warwick University, UK
4. Health Systems Research Unit, Medical Research Council, South Africa

Corresponding author
Jane Goudge
Email: jane.goudge@gmail.com

For peer review only

ABSTRACT

Objective: Community health workers (CHW) are undertaking more complex tasks as part of the move towards universal health coverage in South Africa. CHW are expected to provide promotive and preventative care, make referrals to the local clinic, and follow up on non-attendees. CHW programmes can improve access to care for vulnerable communities, but many such programmes struggle with insufficient supervision. In this paper, we assess the coverage, quality of care, and costs of the service provided by CHW teams with differing configurations of supervisors, some based in formal clinics and some in community health posts.

Participants: CHW, their supervisors, clinic staff, CHW clients.

Methods: We used mixed methods to examine the performance of six CHW teams in vulnerable communities in Sedibeng, South Africa

Results: A CHW had visited 17% of households in the last year, and we estimated they were conducting 1-2 visits per day. At household registration visits, the CHW asked half of the questions required. Respondents remembered 20-25% of the health messages that CHW delivered from a visit in the last month, and half of the respondents took the action recommended by the CHW. Training, supervision and motivation of the CHW and collaboration with other clinic staff were better with a senior nurse supervisor. If CHW carried out four visits a day, coverage would increase to 30-90% of households, suggesting that more CHW are needed in some sites, as well as better supervision.

Conclusion: Household coverage was low, and the service was limited. With skilled senior supervision, the CHW provided care for a broader range of conditions. Support from the local facility was key to providing a quality service, and a senior supervisor facilitated this collaboration. Greater investment in numbers of CHW, supervisors, training and equipment is required for the potential benefits of the programme to be delivered.

Strengths and limitations of this study

For peer review only - http://bmjopen.bmj.com/site/about/guidelines.xhtml

- A wide range of data collection methods enabled triangulation across data sources to provide a more accurate description of the CHW programme.
- The six sites were typical of the Sedibeng District, and while it is not possible to generalise with any certainty, the sites included both rural and peri-urban communities typical of much of South Africa.
- Due to the very low number of respondents reporting a CHW visit in the past month, analyses of disease specific services were not possible.
- During observations, the work effort by the CHW increased due to the presence of fieldworker, although we observed the same CHW over 4 days to reduce this effect.

INTRODUCTION (5000 not including tables, references etc)

Community health workers (CHW) programmes have potential to improve access to care for vulnerable communities[1, 2], and they can be effective in improving health behaviours and outcomes.[3, 4] However, many programmes do not have adequate supervision and resources, resulting in low motivation and poor performance among the CHW, and the expected health benefits do not materialise.[5] With the increasing importance of universal health care coverage, CHW are responsible for a greater range of promotive and preventative care, the skills required are broader and more complex, and the need for supervision is greater.[6] However, due to the shortage of health care workers in low- and middle-income settings, the number of nurses available to supervise CHW is limited. As a result, programme managers often have to choose between locating CHW teams in established clinics, with closer supervision and greater access to resources, or locating teams in health posts away from the clinic but closer to communities in need, enabling a more responsive service and reducing CHW travelling.

South Africa has initiated a national CHW programme (WBOTs; ward-based outreach teams) to strengthen primary health care.[7] The intention is to provide health promotion, prevention, screening and referral for a wide range of health conditions. [8] It is not clear how many households the CHW are able to serve, or whether they are able cope with the broader range of health needs they are meant to provide services for. In deciding how to strengthen and to scale up this programme further, decision-makers need information on the household coverage and quality of care achieved by differing supervisor / location models, as well as the varying costs from the existing teams.

In the initial observation phase of a 3-year intervention study in Sedibeng Health District, we studied CHW teams with different configurations of supervisors and locations: clinic-based teams supervised by a professional nurse (PN) and an enrolled (junior) nurse (EN); health post-based teams supervised by a PN and an EN; and, clinic-based teams supervised by an EN only. In this paper, we report on household coverage, quality and costs of the service of the different models.

METHODS

We used a case study approach, combining qualitative and quantitative data, to examine the operation of six CHW teams, with each team and their supervisors being a single case study.

Study site

In the South African government CHW programme, CHW teams are meant to comprise a nurse, six or more CHW, one health promoter and one environmental officer.[7] In Sedibeng health district (Gauteng Province), at the time of the study, there were 39 CHW teams in 37 of the district's 72 wards (smallest geopolitical area). The teams varied in level of supervision and location of the team's base.

For peer review only - http://bmjopen.bmj.com/site/about/guidelines.xhtml

The supervisors were either professional or enrolled nurses. Professional nurses (PNs) in South Africa are able to diagnose patients, prescribe treatment and dispense medication. The PN supervisors were also trained in primary health care and community nursing. Enrolled nurses (ENs) complete a 2-year nursing course, and are qualified to provide nursing care under supervision.

Sixteen of the teams were based at a health post and the remaining 23 were clinic-based. Some health posts were located relatively close to their 'mother' clinic with the aim of providing outreach services; other health posts were located in communities without a clinic in order to improve access to basic services. A health post consists of one or two temporary wooden structures (providing 3-6 rooms), without electricity and often with irregular water supply. It is managed by one or two PNs, who obtain medication and other resources via a 'mother' clinic. The nurses supervise the CHW team and provide basic services to the community such as chronic medication, immunization and treat minor ailments.

Nation-wide standardized CHW training covers identification of the need for ante- and post-natal care, monitoring immunisation and adherence to chronic medication, screening for malnutrition, TB, gender-based violence, and making referrals to health and social services. In addition, in Sedibeng the CHW deliver chronic medication to elderly, or disabled, patients.

We categorized the CHW teams into the three types and selected two teams of each type (Table 1). We drew on the advice of the district managers, to select each pair of teams to maximise the similarity in the community served by the two teams.

Table 1. Configurations of CHW teams

Model	Supervisor	Based in	Site number
1	Professional & Enrolled Nurse	Clinic	1<br>2
2	Professional & Enrolled Nurse	Health post	3<br>4
3	Enrolled Nurse only	Clinic	5<br>6

Data collection

In each site we conducted a household survey, focus group discussions (FGD) with the CHW, observations of CHWs at work, and interviews with CHWs' clients, CHW supervisors, facility managers and the community representatives from Sept 2016 to Feb 2017 (Table 2).

Table 2: Data collection method, participants and data collected

Component		Participants	Total number in 6 sites	Data collected
Quantitative	Household Survey	Randomly selected households in the catchment area (220 per site)	1227 household interviews	• Socio-economic status of household, demographic profile, access to care, and types of need for care;<br>• Whether a CHW had visited in the past month/year (coverage);<br>• Health messages recalled by the HH (as a proxy for quality of care)
	Costs	Set up and recurrent costs per site	-	Set up and recurrent costs
Qualitative	FDG	CHW teams	12 FGDs (76 participants)	• FGD: Types of activities, challenges of the programme<br>• Self-administered questionnaire: age, years of training and service,<br>• Types of activities carried out; types of clients encountered;<br>• Supervision activities and interaction with clinic staff;
	Obs	CHWs and supervisors while conducting their daily work	126 days of observation	

For peer review only - http://bmjopen.bmj.com/site/about/guidelines.xhtml

	In-depth Interviews	Supervisors, FMs, and community representatives	43 key informant interviews	Perceptions of what the programme entailed, its successes and challenges, whether it was matched with the community needs
		Follow-up interviews with CHW clients who were referred to the clinic during observations	74 household interviews	Whether client took referral action, and outcome client's perception of the service

Household survey

We used stratified sampling (based on area and housing type) to select 220 households per site. The respondent was the person who knew most about the health of household members. Data collection via an electronic device allowed any irregularities to be identified and resolved as the survey progressed. We collected data on coverage (whether a household had been visited in the past year or in the past month), the need for care (number and types of health conditions), and the quality of care provided (the number of health messages recalled for visits in the last month). Health messages are pre-defined advice concerning each condition that the CHW was meant to provide, when relevant. Descriptive statistics were generated for all variables of interest and compared across sites. A logistic regression was conducted to better understand who was receiving CHW services.

Observations, focus group discussions and interviews

Trained fieldworkers were given an orientation to community-based health care by an experienced primary health care nurse. Interview guides and observation templates were revised after internal piloting and feedback from fieldworkers. Respondents, except for CHW, were chosen purposively. A brief survey captured key demographic and career history of the CHW. The selection of CHW to be observed was done randomly (drawing names out of hat) on the first morning of a 4-day observation period. The fieldworkers observed the CHW or supervisor at work and took detailed notes. If a client was given a referral by a CHW, the fieldworkers asked to conduct an interview with the client in a month's time. This subsequent interview provided information on follow up actions taken by the client.

We drew together qualitative data to develop an explanatory description of how each team functioned, and then drew comparisons across teams. Firstly, we extracted data for each site into one document, either summarizing events, or useful quotations as raw data. (Team members extracted data from same sources, compared extracted data and modified extraction strategies until we were confident about inter-extractor reliability.) We presented a brief summary of each site in a one-day workshop, and through discussion identified common and divergent themes across the sites (such as weekly and daily pattern of activities, resources, record keeping, patient referrals to the clinic, engagement with clinic staff, relationship between CHW and supervisors, relationship with patients). We then collated data under these themes, producing a summary for each site (see example in appendix). This allowed us to further compare sites, to see how variation in supervision and integration with the health system influenced the functioning of the teams.

In addition, we counted the observed visits by purpose (medication delivery, follow up visit, registration), which questions were asked during registration, and whether referral action advised by the CHW was taken and reasons why not. (Registration required the completion of a government form identifying nine health conditions/needs: coughing, HIV testing, pregnancy, recent birth, child under five, chronic medication, and need for family planning, home-based care, social grants.)

Costing

We only included the costs to the health system, not household costs, or those incurred by CHWs such as airtime or transport. We included both set up costs (health post infrastructure and equipment, CHW kits, and training), and recurrent costs (staff costs, health post and equipment maintenance, CHW kit replenishment, and overheads). Staff costs included CHWs, supervisors and a share of the responsible district manager. Costs and the estimated life span of infrastructure and

For peer review only - http://bmjopen.bmj.com/site/about/guidelines.xhtml

equipment were collected from the district management team. PHC expenditure per capita for the district was extracted from the Health System Trust District Barometer 2015. All costs are expressed in 2016 Rands after applying adjustment for inflation if required.

All items with an expected life over one year were treated as set-up costs and annualized by expected length of life, using straight depreciation. Costs were derived for each site, based on the particular site's staffing profile. Costs for the district CHW programme were calculated as expenditure per capita. This amount is compared to the per capita PHC expenditure to determine the share of PHC expenditure represented by the CHW programme.

Integrating the qualitative and quantitative evidence

We compared the data on coverage and quality of care from the survey with the qualitative evidence, in order to examine how the different models functioned, and why. We then combined these judgements with the costs to make an assessment about the value for money of the different models.

Ethics

The study was approved by the University of the Witwatersrand's Human Research Ethics Committee (Medical) (M160354), the Gauteng Provincial Health Research Committee, and the Biomedical and Scientific Research Ethics Committee (BSREC) at University of Warwick (REGO-2016-1825). CHW clients and the public were not involved in the design, conduct, reporting or dissemination plans; however, district and provincial managers were involved.

Patient and public involvement

CHW clients and the public were not involved in the design, conduct, reporting or dissemination plans; however, facility, district and provincial managers were involved. We have conducted feedback sessions to CHW teams, facility, district and provincial managers.

RESULTS

The clinics, health posts and the CHW teams

The clinic in Site 1 was a formal, relatively modern building. The clinic in Site 2 was prefabricated wooden structures with limited space, so the CHW had to meet outside. The health post in Site 3 (a repurposed transport container) was located relatively close to its 'mother' clinic. In contrast the health post in Site 4 was located in a community without a clinic. As a result, at this site, the PN supervisor spent half of her time consulting with patients, rather than supervising the CHW. Sites 5 and 6 were both formal clinics in rural settings. Site 6 had sufficient space for CHW to their meetings inside; Site 5 did not and CHW met outside.

The EN supervisors (other than in Site 1) were younger and had less work experience than the CHW. There were no PN supervisors at the two rural sites (sites 5 & 6); the district manager reported it was hard to recruit PNs to work in the rural clinics, because of their location. The size of the teams ranged from 9-20 CHW. More supervision time per CHW was available to the teams in sites 1 and 2, and to a lesser extent Site 3. Using the South African census, we estimated the number of households per pair of CHW varied from 120-400 (Table 3).

The average age of the teams varied between 33-41 years, and the percentage who had completed their matric ranged from 25% to 63%. Length of time working as CHW ranged from 5-10 years. The majority of CHW had completed Phase 1 WBOT training (90-100%), except for the two rural sites (sites 5 &6) that were located over an hour's drive from the training facility.

The CHWs often worked without necessary resources (equipment, stationery, uniform, or funds for transport or mobile communication). The CHWs were meant to have a bag of equipment, which

For peer review only - http://bmjopen.bmj.com/site/about/guidelines.xhtml

included a blood pressure machine, a glucometer and testing strips, a weighing scale, MUAC tape, bandages, gloves, mask, forms, raincoat and water bottle. At the start of the fieldwork, many CHW were without bags. Equipment bags were delivered during the data collection but were not regularly re-stocked. In one team the CHW had not received training on how to use the manual blood pressure machines provided. The notebook and pen that CHW used to record daily activities had to be purchased out of their stipend, and so many of them did not have these items during our observations. They used pieces of papers to record the visits. On occasions, CHWs did not have the household registration form because the photocopier was broken. Two of the six teams did not have sufficient space to complete paperwork and to store their files; often files were kept at home.

The CHW were not formally employed by the government. They were an outsourced workforce and paid a minimal stipend for 6 hours work per day. CHWs struggled to contact the private payroll company responsible for monitoring attendance and paying stipends. An electronic system, requiring CHWs to clock in/out at the clinic, limited their ability to reach outlying areas. Two months prior to the start of our fieldwork CHW had been on strike over their conditions of employment. During the fieldwork the CHW were paid (2500 RSA), below the minimum wage (R3500 per month), however, by the end of 2018, it was increased to the minimum wage.

Household characteristics, need for, and access to, care

The proportion of households who agreed to participate in the survey ranged from 85-97% across the sites. Site 1 contained mostly formal dwellings, all of which had access to piped water inside their house (Table 3). Site 4 was predominately an informal settlement, with 3.8% of households having piped water inside the house. In Site 5, half of the dwellings were informal. Access to the internet ranged from 25 – 42% of households, with 31% of households having access in the informal settlement (Site 4). The age distribution of the population is similar with the informal settlements (Sites 4 & 5) having fewer children under 5, and the more formal settlement (Site 1) having double the number of people aged 60 and over. The need for care was greatest in the informal settlements; in Site 5, 19% of households reported 3 different conditions or need for care due to high levels of HIV and TB. The formal settlement (Site 1) had higher levels of hypertension and diabetes due to its elderly population.

Household coverage

Across the sites 10-20% of households had been visited in the last year, with 5-12% visited in the last month (Table 4). Having only an EN supervisor was associated with a higher level of coverage. Those households with a person aged 60 and above were also almost twice as likely to receive a visit in the past year (due to the delivery of medication to elderly patients). However, other characteristics that we would expect to increase the likelihood of a visit, such as a child under 5, the number of health care needs, and dwelling type (a proxy for socio-economic status), did not. CHW were not more likely to visit households closer to the clinic or health post. Combining the household survey data with the estimated number of households in each catchment area, we estimated that a pair of CHW on average visited between 1-2 households per day. However, during the observations, the CHW visited on average of 3-5 households per CHW pair per day.

Type, and quality, of care delivered

The purpose of household visits varied. CHW delivered medication in nearly half of observed visits (47%), followed up with a patient (38%) or registered a household not visited before (15%). None of the observed household registrations were fully completed, with CHWs completing on average 4-5 of the 9 questions (Table 5). The three most frequently asked about health conditions / needs were: a children under five, a person taking daily medication and a birth in the last 6 weeks (in >70% of registrations). The need for HIV testing and family planning was established in at least half of registration visits. Coughing, the need for social grants and pregnancy status were the least discussed. In a third of visits an additional health need was established, as CHWs asked about other health issues, although this varied across sites (72.5% in Site 4 and 18.2% in Site 3).

For peer review only - http://bmjopen.bmj.com/site/about/guidelines.xhtml

The survey respondents in sites 1 and 2 (with PN and EN supervisors) recalled a quarter of the relevant messages for the conditions discussed during the visit in the last month (Table 5). However, respondents in sites 4 (health post team in a community without access to a clinic) and 6 (one of the EN-only sites) recalled only 11% and 13% of the relevant messages.

Half of the clients sought care at the clinic when referred by the CHW. The most common reason given for no action was lack of funds or transport. In Site 6 only 39% of referrals were acted-on. Key community informants reported complaints had been made about staff attitudes at the clinic, which may have influenced whether the patient took action.

Supervision practices and support from the health system

We observed experienced supervisors using job training, supervised household visits, and debriefing sessions (during which problems were discussed) to train, motivate and monitor CHW, and improve the quality of their work. In one site, the PN gave brief education sessions about common health conditions in the community before the CHW left the clinic in the morning, "*I cover hypertension, diabetes, HIV, the children's Road to Health card, TB, health hazards, prostate and cervical cancer screening. It is things they deal with in the community.*" (PN, Site 1). One health post-based PN/EN team (Site 3) held daily debriefing sessions, at which CHWs described the households visited, problems encountered, and actions taken. The sessions strengthened CHWs' knowledge base and problem-solving abilities, and provided a platform for collective supervision, enabling the CHWs to learn from each other's experience. Those teams that had no place to meet inside the facility tended not to discuss serious matters for reasons of confidentiality. *"Sometimes we stand outside to hold meetings… patients and the security personnel will be listening to what we are discussing." (CHW-FGD, Site 5)*

ENs routinely accompanied different pairs of CHWs on home visits several days a week. One EN (who worked as part of an EN/PN pair) demonstrated sensitivity in correcting CHWs' practice. *"When I can see this one didn't do it right, I keep quiet in the house, but immediately after we step outside as we are walking, I do on-the-spot training" (EN, Site 2)*. Despite one EN (without a PN supervisor) spending 4 days a week out in the community, the CHWs did not acknowledge her as a supervisor, *"When we get there she's doing the same job that I normally do when I am alone." (CHW-FGD, Site 5)*

Job satisfaction, professional confidence and motivation were more evident in teams with a PN supervisor. One CHW commented that since the PN arrived: *"we started to feel that we are now CHWs and human beings." (CHW-FGD, Site 1)* When the CHWs were on strike over their conditions of employment, some of the CHWs at the clinic-based PN/EN Site (Site 2) tried to carry on, not wanting to let their patients down or damage their relationship with supervisors and the facility. Their supervisor interpreted their actions: *"they see that you, as their leader, are supporting them and trying to understand where they come from, especially with this employer thing being mixed up. That is why I am saying, hence, they refused to go on strike today." (EN, Site 2)*

However, in the absence of supportive supervision in EN only sites, insufficient training constrained CHWs' ability to assist patients: '*since we work under WBOT we have not been trained,*" *(FGD Site 6).* Based an hour's drive from the district office, the rural sites rarely received training. With their frustrations over their unresolved working conditions, which were not acknowledged by their immediate supervisors, the CHW were demotivated: "*the way the contractor is operating, they make us to be lazy, because since we signed a contract with them, we have never seen them or had a meeting with them to tell us about their rules*" *(FGD Site 6).* Field notes also showed that many CHW did not make full use of their time due to a lack of motivation and ineffective time-task management. Some CHWs decided to work fewer hours a day as a passive protest.

For peer review only - http://bmjopen.bmj.com/site/about/guidelines.xhtml

In contrast, in the clinic-based teams, with the support of PN supervisors, and the resulting growth in skills and confidence, CHWs had been able to establish better relationships with clinic staff. In turn, clinic staff, seeing the CHW's greater motivation and effort, were more likely to treat the CHWs with respect. Communication and coordination was better. Working together, the facility manager and PN were able to confirm whether, once defaulters had been traced by CHWs, they returned to the clinic. *"We engage with facility manager, she helps with problems that we have... we include her in everything" (EN, Site 2)*. Clinic staff willingly accepted referrals from CHWs, and included the CHWs in training sessions: *"Sister X is not (in the CHW team), but she is very helpful. She attended severe malnutrition training and she called all the CHWs and did a presentation for everybody*." *(EN, Site 2)*. As a result the contribution of CHWs was highly valued: *"If they (CHWs) were not there, it means you will be over worked, the clinic will always be full, to be honest. There is good communication between us." (Facility Manager, Site 1)*. Located at a distance from the main clinic, one health post team (*Site 4*) struggled to engage with clinic staff, and to ensure patients accessed to care at the clinic. (For a full description of the supervisory practices, please refer to our companion paper.[9])

Costs

Across the district as a whole the annual expenditure on the WBOT programme was R44 million (2016 Rands), at an average cost per CHW of R52,000 (2016 Rands). The cost per capita (for the district population without private health insurance) was R47. District expenditure per capita on primary health care (including community health centres and clinics, but not district hospitals) was R1,200. We therefore estimated that WBOT expenditure in the district was only 3.9% of PHC expenditure.

The cost per CHW are higher the smaller the number of CHW per supervisor, as well as with the start-up costs of the health post (Table 5). The cost per CHW of the EN-only model in the clinic (Sites 5 & 6) was lowest; although this model achieved higher coverage, the observation data suggests the care provided was poorer. Site 3 provided high quality care, but was also expensive, because of the small number of CHW and cost of the health post. The PN & EN model based in the clinic (Sites 1 &2) provided better quality of care due to the PN supervisor and the support of the clinic, at a lower cost as there was no health post set up costs.

For peer review only

TABLE 3: Description of catchment area, households and members surveyed, and their need for care		PN & EN BASED IN CLINIC				PN & EN BASED IN HEALTH POST				EN ONLY BASED IN CLINIC			
		Site 1		Site 2		Site 3		Site 4		Site 5		Site 6	
**2016 DEPARTMENT OF HEALTH DISTRICT DATA**													
**Catchment area**	Population in CHW catchment area (N)	23,877		6,749		10,984		5,000		5,838		16,610	
	HH per Site (N)	6,453		2,177		3,328		1,429		1824		4,746	
	No of HH per CHW pair	403		128		396		119		130		237	
	No of people per household (Mean)	3.7		3.1		3.3		3.5		3.2		3.5	
**HOUSEHOLDS SURVEYED (n)**		**187**		**186**		**224**		**213**		**209**		**206**	
**SES, access to, and need for, care**	HH informal dwelling (%, n)	0.0	0	25.8	48	13.5	30	96.2	205	43.1	90	23.7	47
	Access to piped water indoors (%, n)	54.6	102	39	73	53.6	120	3.8	8	44.0	92	64.6	133
	Access to internet (yes) (%, n)	42.3	79	25.8	48	33.9	76	31.0	66	25.4	53	32.5	67
	Walk to the clinic (yes) (%, n)	77.4	144	63.4	129	88.8	199	74.7	159	89.0	186	87.3	179
	Distance from house to clinic / health post (km) (median, IQR)	0.7	0.5-1.0	1.7	1.5-2.2	0.4	0.2-0.7	0.4	0.3-0.6	2.4	2.0-2.9	0.6	0.4-1.3
	3 or more health conditions or needs (%, n)	18.2	34	12.4	23	11.6	26	16.4	35	19.6	41	14.1	29
**INDIVIDUALS SURVEYED (n)**		**692**		**574**		**747**		**755**		**679**		**722**	
**Age**	0-4 (%, n)	6.5	45	7.8	45	7.9	59	3.8	29	4.3	29	7.9	57
	5-18 (%, n)	19.4	134	26.7	153	26.9	201	26.8	202	25.9	176	28.5	206
	19-59 (%, n)	58.7	406	59.6	342	58.5	437	61.6	465	63.0	428	56.1	405
	60+ (%, n)	15.5	107	5.9	34	6.7	50	7.8	59	6.8	46	7.5	54

Note: Distance = direct distance between GPs co-ordinates

For peer review only

BMJ Open

Table 4: Household coverage and quality of care

		PN & EN BASED IN CLINIC				PN & EN BASED IN HEALTH POST				EN ONLY BASED IN CLINIC			
		Site 1		Site 2		Site 3		Site 4		Site 5		Site 6	
**Coverage**	**All households SURVEYED (n)**	**187**		**186**		**224**		**213**		**209**		**206**	
	HH visited by CHW in the last year (%, n)	10.2	19	11.8	22	15.6	35	16.0	34	19.6	41	19.9	41
	HH visited by CHW in the last month (%, n)	5.3	10	7.5	14	8.9	20	6.6	14	12.4	26	5.8	12
**Purpose of visit, whether registration completed or additional need**	**Household visits OBSERVED (n)**	**81**		**87**		**77**		**40**		**89**		**128**	
	Household Registration (%, n)	27.1	22	6.8	6	-		72.5	29	-		16.4	21
	Medicine delivery (%, n)	66.7	54	48.3	42	59.7	46	20.0	8	49.4	44	33.6	43
	Follow up (e.g. missed clinic appt, hospital discharge) (%, n)	6.2	5	44.8	39	42.8	33	7.5	3	50.6	45	51.6	66
	Average number of registration questions asked (%, n)	53.5	4.8/9	55.5	5/9	-		50.2	4.5/9	-		43.9	4/9
	Visits whereby CHW found a need (%, n)	60.0	49	27.5	24	18.2	14	72.5	29	19.1	17	35.9	46
**Advice recalled**	**All households SURVEYED (n)**	**187**		**186**		**224**		**213**		**209**		**206**	
	Proportion of relevant health messages recalled (ratio, SE)	0.27	0.03	0.26	0.03	0.18	0.03	0.13	0.04	0.20	0.02	0.13	0.07
	Proportion of messages recalled for hypertension (ratio, SE)	0.28	0.02	0.27	0.03	0.21	0.04	0.12	0.05	0.24	0.02	0.19	0.09
**Referrals acted upon**	**Total individual interactions during OBSERVED HH visits (n)**	**127**		**128**		**112**		**79**		**136**		**165**	
	Number of referrals recorded (%, n)	12.6	16	13.3	17	17.0	19	15.2	12	19.1	26	10.3	17
	Number of referred individuals interviewed (%, n)	-	14	-	13	-	15	-	6	-	16	-	13
	Patients who took referral action (%, n)	57	8	54	7	53	8	50	3	69	11	39	5

For peer review only

Table 5: Annualized costs of the different models

RANDS 2016	PN & EN BASED IN CLINIC		PN & EN BASED IN HEALTH POST		EN ONLY BASED IN CLINIC	
	**Site 1**	**Site 2**	**Site 3**	**Site 4**	**Site 5**	**Site 6**
**Number of PN supervisors**	1	1	1	1	0	0
**Number of EN supervisors**	2	2	1	1	1	1
**Total costs per team**	1,047,953	1,083,809	938,344	910,240	694,429	909,564
**Number of CHW**	16	17	9	12	14	20
**Cost per CHW**	65,497	63,753	104,260	73,853	49,602	45,478
**Explanation for variation in costs**	Two ENs in each of these sites, so total costs are higher, but the teams are larger so supervision is needed		Fewer CHW per supervisor so more expensive, as well as the costs of the health post	Larger team so cheaper than Site 3, but one supervisor actually consulting patients	With a fewer supervisor and larger teams, the costs were lower	
**Quality of care**	High quality		High quality	Poorer quality because less supervision and significant distance to facility	Poor quality, because insufficient supervision	
**Value for money**	Good value: good supervision, and well integrated into the health system, even in clinic where there is not enough space for the CHW		High quality model, but intensive because few CHW, and so expensive.	In mid-range in terms of expense but trying to provide basic clinic services as well, so quality suffers	Poor value: Cheaper but poor quality, ineffective care	

For peer review only

DISCUSSION

Household coverage was limited in all the sites (10-20%). The low proportion of registration visits suggests that the CHW were revisiting households they know (driven by the requests by the clinic to deliver medication or trace patients who had stopped attending the clinic) rather than taking on the more difficult task of approaching an unknown household, and so limiting the number of households they were responsible for. Insufficient training and resources demotivated the CHW and generated resentment. Our study suggests that the CHW could achieve a greater number of visits per day, and that more effort could be made towards targeting households in greater need (e.g. those with young children). An increase to four visits per CHW pair per day would achieve coverage ranging from 30-90% (the variation is due to the number of households and CHW).

CHW did not ask all the registration questions; without knowing the full range of health and social needs of the households, they are unlikely to provide the necessary care. However, the CHW identified additional need(s) (beyond the purpose of the visit) in a third of households. Household respondents remembered between a fifth to a quarter of the health messages, and half of patients took the referral action recommended by the CHW.

Where there was better, more senior supervision, household members were more likely to recall the advice and messages given; where there is less supervision and less training, the CHW are visiting more households, but providing poorer quality care. Teams based in a clinic, with a senior supervisor, are more likely to be better integrated into the health system, and so able to provide a higher quality service. Without senior supervision the EN supervised teams, although located in a clinic, were not integrated into the system as they didn't receive the necessary training or on-going support, and they struggled to negotiate effective working relationships with other facility staff members. In health posts that served a community without a clinic, nurse supervisors had to consult directly with patients arriving and had less time to supervise the CHWs, and engagement with the clinic was difficult due to distance.

In this study, district expenditure on the CHW programme is only 3.9% of expenditure on non-hospital primary health care. Greater investment in more capable supervision, and where necessary, a greater number of CHW, would increase coverage, and improve the quality of the service. Box 1 sets out our recommendations from this phase of the study. Given the shortage of professional nurses, scaling up the PN & EN model is not feasible, so cost-effective ways need to be found to train and provide on-going to support to the EN supervisors in order to improve the quality of their supervision. In the second phase of this study, we are evaluating at an intervention to provide mentorship to the EN supervisors in sites 5 &6.

Due to the very low number of respondents reporting a CHW visit in the past month, analyses of specific services were not possible. As a result, we have relied on the household registration visits to assess the comprehensiveness of the service provided. During observations, the work effort by the CHW increased due to the presence of fieldworker. We observed the same CHW over 4 days in order to reduce this effect.

The study's strength is the wide range of data collection methods that enabled triangulation across the different data sources, including the observation of CHW activity rather than self-reported data. The six sites were typical of the Sedibeng District, and while it is not possible to generalise with any certainty, the sites included both rural and peri-urban communities typical of much of the country.

Most evaluations of CHW programmes are focused either on a single condition, or cluster of conditions (e.g. maternal and child health); there is limited research assessing the performance of CHW in more comprehensive programmes. Where delivery of care is focused on maternal and child

For peer review only

health, CHWs have been found to struggle with providing comprehensive care due to insufficient supervision and support from the associated health service [10][11]

Several recent studies have focused on the degree of integration of CHW with the health system, identifying barriers such as resistance from other health workers, discrimination against CHW based on social, gender and economic status, ineffective incentives, inadequate supplies and infrastructure [12]. Lawn et al provides evidence on cost suggesting need for increased coverage, and mentions the issue of how much CHW are paid. [13] However, there is little research on both household coverage and the quality of care provided; this is important as there is a potential trade-off between the two. In addition, there is little on evidence the synergistic effect of senior supervision, which may be important in enabling integration with the health system, and improving both coverage and quality of care.

Box 1: Recommendations for an effective CHW programme

1. Dedicated, experienced supervision (both to build CHWs', and their supervisors skills and to facilitate collaboration with the closest facility) is essential otherwise the investment in CHW programme is unlikely to achieve the household coverage or quality of care possible.
2. Where there are insufficient numbers of CHW, the programme needs to comprise a larger proportion of the PHC budget, in order to substantially increase the coverage of the programme;
3. A room inside the clinic to meet and compile reports is a necessary but not sufficient for the integration between clinic and the CHW team. Where physically not possible, an additional structure should be build next to facility
4. Health posts in locations without a formal clinic to serve the community should be converted into formal clinics, or at least provided with sufficient resources and infra-structure so that they can operate as a full clinic.

CONCLUSION

The CHW provided their service to less than a fifth of households in the catchment communities. The service the CHW provided to the households they visited was limited. Where skilled senior supervision was available, the CHW were able to provide a service that covered a broader range of health needs. Support from the local facility for the CHW team is key to providing a quality service, and a senior supervisor can help to facilitate this collaboration. Greater investment in numbers of CHW, supervisors, training and equipment is required for the potential benefits of the programme to be delivered.

Acknowledgements

We would like to thank all of the study participants, as well as district and provincial management for their support and engagement with the study

Funding Acknowledgements

The study was funded by Medical Research Council UK, DFID, ESRC, and Wellcome Trust under the Joint Health Systems Research Initiative

For peer review only - http://bmjopen.bmj.com/site/about/guidelines.xhtml

Author contributions
JG, MT, FG conceptualised the study and raising the funding.
JG lead the drafting of the manuscript.
JG, JDK, OB, HM, were responsible for overseeing data collection
MM, JL, JDK, OB were responsible for statistical analysis
JG, FG, HM MK, TR, NN, JAW were responsible for qualitative analysis
JG, FG, HM, MK, TR, NN, JAW, MT, OB contributed to the drafting the manuscript
All authors have read the final draft.

Author characteristics
JG is Professor of Health Policy and Systems at Centre for Health Policy
JdK during the study was a researcher / project manager at the Centre for Health Policy
OB is researcher / project manager at the Centre for Health Policy
MM during the study was a bio-statistican at School of Public Health
MK during the study was a post-doctoral fellow at Centre for Health Policy
HM & TR are PhD fellows at Centre for Health Policy
NN is a senior researcher and Deputy Director at the Centre for Health Policy
JL is Professor of Biostatistics at School of Public Health
MT is Emeritus Professor at Warwick Medical School
ED during the study was a health economist at the South African Medical Research Council
JAW is a researcher at Warwick Medical School
FG is Professor of Medicine and Society at Warwick Medical School
None of the authors had any prior interaction with the respondents.

Data
Quantitative data can be made available on request. Given the difficult of anonymizing qualitative, we will work with any researchers wishing to further analyse the qualitative data.

Declaration of Conflicting Interests
Nothing to declare.

References

1. Zulliger, R., M. Moshabela, and H. Schneider, *"She is my teacher and if it was not for her I would be dead": Exploration of rural South African community health workers' information, education and communication activities.* Aids Care-Psychological and Socio-Medical Aspects of Aids/Hiv, 2014. **26**(5): p. 626-632.
2. Walley, J., et al., *Alma-Ata: Rebirth and revision 8 - Primary health care: making Alma-Ata a reality.* Lancet, 2008. **372**(9642): p. 1001-1007.
3. Christopher, J.B., et al., *Thirty years after Alma-Ata: a systematic review of the impact of community health workers delivering curative interventions against malaria, pneumonia and diarrhoea on child mortality and morbidity in sub-Saharan Africa.* Human Resources for Health, 2011. **9**.
4. Glenton, C., et al., *Can lay health workers increase the uptake of childhood immunisation? Systematic review and typology.* Tropical Medicine and International Health, 2011. **16**(9): p. 1044-1053.
5. Pallas, S.W., et al., *Community Health Workers in Low- and Middle-Income Countries: What Do We Know About Scaling Up and Sustainability?* American Journal of Public Health, 2013. **103**(7): p. E74-E82.
6. Tulenko, K., et al., *Community health workers for universal health-care coverage: from fragmentation to synergy.* Bulletin of the World Health Organization, 2013. **91**(11): p. 847-852.

For peer review only

7. SADoH. *Provincial guidelines for the implementation of the three streams of PHC Re-engineering*. 2011 [cited 2017 23/January/2017]; Available from: http://www.rmchsa.org/wp-content/resources/resources_by_type/DistrictLevelResources/ProvincialGuidelines_PrimaryHealthCare3StreamsOverview2011.pdf.
8. Schneider, H., et al., *The challenges of reshaping disease specific and care oriented community based services towards comprehensive goals: a situation appraisal in the Western Cape Province, South Africa.* BMC health services research, 2015. **15**(1): p. 436.
9. Tseng, Y.H., et al., *Integrating community health workers into the formal health system to improve performance: a qualitative study on the role of on-site supervision in the South African programme.* BMJ Open, 2019. **9**(2): p. e022186.
10. Wilford, A., et al., *Exploring the care provided to mothers and children by community health workers in South Africa: missed opportunities to provide comprehensive care.* BMC public health, 2018. **18**(1): p. 171.
11. Haskins, J.L., et al., *Fragmentation of maternal, child and HIV services: A missed opportunity to provide comprehensive care.* African Journal of Primary Health Care & Family Medicine, 2016. **8**(1): p. 1240.
12. Zulu, J.M., et al., *Integrating national community-based health worker programmes into health systems: a systematic review identifying lessons learned from low-and middle-income countries.* BMC Public Health, 2014. **14**: p. 987.
13. Lawn, J.E., et al., *Cadres, content and costs for community-based care for mothers and newborns from seven countries: implications for universal health coverage.* Health Policy and Planning, 2017. **32**(suppl_1): p. i1-i5.

For peer review only

Standards for Reporting Qualitative Research checklist

No	Topic	Item	Whether included in the manuscript
S1	Title	Concise description of the nature and topic of the study Identifying the study as qualitative or indicating the approach (e.g., ethnography, grounded theory) or data collection methods (e.g., interview, focus group) is recommended	Yes
S2	Abstract	Summary of key elements of the study using the abstract format of the intended publication; typically includes background, purpose, methods, results, and conclusions	Yes
**Introduction**			
S3	Problem formulation	Description and significance of the problem/phenomenon studied; review of relevant theory and empirical work; problem statement	Yes
S4	Purpose or research question	Purpose of the study and specific objectives or questions	yes
**Methods**			
S5	Qualitative approach and research paradigm	Qualitative approach (e.g., ethnography, grounded theory, case study, phenomenology, narrative research) and guiding theory if appropriate; identifying the research paradigm (e.g., postpositivist, constructivist/ interpretivist) is also recommended; rationale	Yes
S6	Researcher characteristics and reflexivity	Researchers' characteristics that may influence the research, including personal attributes, qualifications/experience, relationship with participants, assumptions, and/or presuppositions; potential or actual interaction between researchers' characteristics and the research questions, approach, methods, results, and/or transferability	Yes; Research characteristics are listed at the end of the article
S7	Context	Setting/site and salient contextual factors; rationale	Yes
S8	Sampling strategy	How and why research participants, documents, or events were selected; criteria for deciding when no further sampling was necessary (e.g., sampling saturation); rationale	Yes
S9	Ethical issues pertaining to human subjects	Documentation of approval by an appropriate ethics review board and participant consent, or explanation for lack thereof; other confidentiality and data security issues	Yes
S10	Data collection methods	Types of data collected; details of data collection procedures including (as appropriate) start and stop dates of data collection and analysis, iterative process, triangulation of sources/methods, and modification of procedures in response to evolving study findings; rationale	Yes
S11	Data collection instruments and technologies	Description of instruments (e.g., interview guides, questionnaires) and devices (e.g., audio recorders) used for data collection; if/how the instrument(s) changed over the course of the study	Yes
S12	Units of study	Number and relevant characteristics of participants, documents, or events included in the study; level of participation (could be reported in results)	Yes
S13	Data processing	Methods for processing data prior to and during analysis, including transcription, data entry, data management and security, verification of data integrity, data coding, and anonymization/deidentification of excerpts	Yes
S14	Data analysis	Process by which inferences, themes, etc., were identified and developed, including the researchers involved in data analysis; usually references a specific paradigm or approach; rationale	Yes
S15	Techniques to enhance trustworthiness	Techniques to enhance trustworthiness and credibility of data analysis (e.g., member checking, audit trail, triangulation); rationale	Yes
**Results**			
S16	Synthesis and interpretations	Main findings (e.g., interpretations, inferences, and themes); might include development of a theory or model, or integration with prior research or theory	Yes
S17	Links to empirical data	Evidence (e.g., quotes, field notes, text excerpts, photographs) to substantiate analytic findings	Yes
**Discussion**			
S18	Integration with prior work, implications, transferability and contribution to the field	Short summary of main findings; explanation of how findings and conclusions connect to, support, elaborate on, or challenge conclusions of earlier scholarship; discussion of scope of application/ generalizability; identification of unique contribution(s) to scholarship in a discipline or field	Yes
S19	Limitations	Trustworthiness and limitations of findings	Yes
**Other**			
S20	Conflicts of interest	Potential sources of influence or perceived influence on study conduct and conclusions; how these were managed	Yes

For peer review only

S21	Funding	Sources of funding and other support; role of funders in data collection, interpretation, and reporting	Yes

For peer review only

BMJ Open

Household coverage, quality and costs of care provided by community health worker teams and the determining factors: Findings from a mixed methods study in South Africa

Journal:	*BMJ Open*
Manuscript ID	bmjopen-2019-035578.R1
Article Type:	Original research
Date Submitted by the Author:	15-Apr-2020
Complete List of Authors:	Goudge, Jane; University of the Witwatersrand School of Public Health, Centre for Health Policy<br>de Kadt, Julia; University of the Witwatersrand, Gauteng City-Region Observatory<br>Babalola, Olukemi; University of the Witwatersrand School of Public Health, Centre for Health Policy<br>Muteba, Michel; University of the Witwatersrand, School of Public Health, Faculty of Health Sciences<br>Tseng, Yu-hwei; University of the Witwatersrand, Centre for Health Policy, School of Public Health, Faculty of Health Sciences<br>Malatji, Hlologelo; University of the Witwatersrand School of Public Health, Centre for Health Policy; University of the Witwatersrand Centre for Health Policy,<br>Rwafa, Teurai; University of the Witwatersrand, Centre for Health Policy, School of Public Health, Faculty of Health Sciences<br>Nxumalo, Nonhlanhla; University of the Witwatersrand, Centre for Health Policy; Centre for Health Policy<br>Levin, Jonathan; University of the Witwatersrand, School of Public Health, Faculty of Health Sciences<br>Thorogood, Margaret; University of Warwick, Medical School<br>Daviaud, Emmanuelle; South African Medical Research Council, Health Systems<br>Watkins, Jocelyn ; University of Warwick,<br>Griffiths, Frances; University of Warwick, Warwick Medical School
**Primary Subject Heading**:	Health services research
Secondary Subject Heading:	Global health, Health policy
Keywords:	Organisation of health services < HEALTH SERVICES ADMINISTRATION & MANAGEMENT, Quality in health care < HEALTH SERVICES ADMINISTRATION & MANAGEMENT, Human resource management < HEALTH SERVICES ADMINISTRATION & MANAGEMENT

I, the Submitting Author has the right to grant and does grant on behalf of all authors of the Work (as defined in the below author licence), an exclusive licence and/or a non-exclusive licence for contributions from authors who are: i) UK Crown employees; ii) where BMJ has agreed a CC-BY licence shall apply, and/or iii) in accordance with the terms applicable for US Federal Government officers or employees acting as part of their official duties; on a worldwide, perpetual, irrevocable, royalty-free basis to BMJ Publishing Group Ltd ("BMJ") its licensees and where the relevant Journal is co-owned by BMJ to the co-owners of the Journal, to publish the Work in this journal and any other BMJ products and to exploit all rights, as set out in our licence.

The Submitting Author accepts and understands that any supply made under these terms is made by BMJ to the Submitting Author unless you are acting as an employee on behalf of your employer or a postgraduate student of an affiliated institution which is paying any applicable article publishing charge ("APC") for Open Access articles. Where the Submitting Author wishes to make the Work available on an Open Access basis (and intends to pay the relevant APC), the terms of reuse of such Open Access shall be governed by a Creative Commons licence – details of these licences and which Creative Commons licence will apply to this Work are set out in our licence referred to above.

Other than as permitted in any relevant BMJ Author's Self Archiving Policies, I confirm this Work has not been accepted for publication elsewhere, is not being considered for publication elsewhere and does not duplicate material already published. I confirm all authors consent to publication of this Work and authorise the granting of this licence.

For peer review only

Title: Household coverage, quality and costs of care provided by community health worker teams and the determining factors: Findings from a mixed methods study in South Africa

Authors: Jane Goudge[1], Julia de Kadt[1], Olukemi Babalola[1], Michel Muteba[2], Yu-hwei Tseng[1], Hlologelo Malatji[1], Teurai Rwafa[1], Nonhlanhla Nxumalo[1], Jonathan Levin[2], Margaret Thorogood[1,3], Emmanuelle Daviaud[4], Jocelyn Anstey Watkins[3], Frances Griffiths[1, 3].

Affiliations

1. Centre for Health Policy, School of Public Health, Faculty of Health Sciences, University of the Witwatersrand, Johannesburg
2. School of Public Health, Faculty of Health Sciences, University of the Witwatersrand, Johannesburg
3. Warwick Medical School, Warwick University, UK
4. Health Systems Research Unit, Medical Research Council, South Africa

Corresponding author
Jane Goudge
Email: jane.goudge@gmail.com

ABSTRACT

Objective: Community health workers (CHW) are undertaking more complex tasks as part of the move towards universal health coverage in South Africa. CHW programmes can improve access to care for vulnerable communities, but many such programmes struggle with insufficient supervision. In this paper, we assess coverage (proportion of households visited by a CHW in the past year & month), quality of care, and costs of the service provided by CHW teams with differing configurations of supervisors, some based in formal clinics and some in community health posts.

Participants: CHW, their supervisors, clinic staff, CHW clients.

Methods: We used mixed methods (a random household survey, focus group discussions, interviews and observations of the CHW at work) to examine the performance of six CHW teams in vulnerable communities in Sedibeng, South Africa

Results: A CHW had visited 17% of households in the last year, and we estimated they were conducting 1-2 visits per day. At household registration visits, the CHW asked half of the questions required. Respondents remembered 20-25% of the health messages that CHW delivered from a visit in the last month, and half of the respondents took the action recommended by the CHW. Training, supervision and motivation of the CHW, and collaboration with other clinic staff, were better with a senior nurse supervisor. We estimated that if CHW carried out four visits a day, coverage would increase to 30-90% of households, suggesting that some teams need more CHW, as well as better supervision.

Conclusion: Household coverage was low, and the service was limited. Support from the local facility was key to providing a quality service, and a senior supervisor facilitated this collaboration. Greater investment in numbers of CHW, supervisors, training and equipment is required for the potential benefits of the programme to be delivered.

Strengths and limitations of this study

- A wide range of data collection methods enabled triangulation across data sources to provide a more accurate description of the CHW programme.

For peer review only - http://bmjopen.bmj.com/site/about/guidelines.xhtml

- The six sites were typical of the Sedibeng District, and while it is not possible to generalise with any certainty, the sites included both rural and peri-urban communities typical of much of South Africa.
- Due to the very low number of respondents reporting a CHW visit in the past month, analyses of disease specific services were not possible.
- During observations, the work effort by the CHW increased due to the presence of fieldworker, although we observed the same CHW over 4 days to reduce this effect.

INTRODUCTION (5000 not including tables, references etc)
Community health workers (CHW) programmes have the potential to improve access to care for vulnerable communities[1, 2], and they can be effective in improving health behaviours and outcomes.[3, 4] However, many programmes do not have adequate supervision and resources, resulting in low motivation and poor performance among the CHW, and the expected health benefits do not materialise.[5] With the increasing importance of universal health care coverage, CHW are responsible for a greater range of promotive and preventative care, the skills required are broader and more complex, and the need for supervision is greater.[6] However, due to the shortage of health care workers in low- and middle-income settings, the number of nurses available to supervise CHW is limited.[7] [8]As a result, programme managers often have to choose between locating CHW teams in established clinics, with closer supervision and greater access to resources, or locating teams in health posts away from the clinic but closer to communities in need, enabling a more responsive service and reducing CHW travelling.

South Africa initiated a national CHW programme (called ward-based outreach teams) in 2011 to strengthen primary health care.[9] The intention is to provide health promotion, prevention, screening services and referral for a wide range of health conditions. [10] While policy documents suggested that CHW should care for approximately 150-250 households,[11] it is not clear how many households the CHW are able to serve, or whether they are able cope with the broader range of health needs they are meant to provide services for. In deciding how to strengthen and to scale up this programme further, decision-makers need information on the household coverage and quality of care achieved by differing supervisor / location models, as well as the varying costs from the existing teams.

In the initial observation phase of a 3-year intervention study in Sedibeng Health District, we studied CHW teams with different configurations of supervisors and locations: clinic-based teams supervised by a professional nurse (PN) and an enrolled (junior) nurse (EN); health post-based teams supervised by a PN and an EN; and, clinic-based teams supervised by an EN only. In this paper, we report on household coverage, quality and costs of the service of the different models.

METHODS

We used a case study approach[12], combining qualitative and quantitative data, to examine the operation of six CHW teams, with each team and their supervisors being a single case study.

Study site

In the South African government CHW programme, CHW teams are meant to comprise a nurse, six or more CHW, one health promoter and one environmental officer.[9] In Sedibeng health district (Gauteng Province), at the time of the study, there were 39 CHW teams in 37 of the district's 72 wards (smallest geopolitical area). The teams varied in level of supervision and location of the team's base.

The supervisors were either professional or enrolled nurses. Professional nurses (PNs) in South Africa are able to diagnose patients, prescribe treatment and dispense medication. The PN supervisors were

For peer review only - http://bmjopen.bmj.com/site/about/guidelines.xhtml

also trained in primary health care and community nursing. Enrolled nurses (ENs) complete a 2-year nursing course, and are qualified to provide nursing care under supervision.

Sixteen of the teams were based at a health post and the remaining 23 were clinic-based. Some health posts were located relatively close to their 'mother' clinic with the aim of providing outreach services; other health posts were located in communities without a clinic in order to improve access to basic services. A health post consists of one or two temporary wooden structures (providing 3-6 rooms), without electricity and often with irregular water supply. It is managed by one or two PNs, who obtain medication and other resources via a 'mother' clinic. The nurses supervise the CHW team and provide basic services to the community such as chronic medication, immunization and treat minor ailments.

Nation-wide standardized CHW training covers identification of the need for ante- and post-natal care, monitoring immunisation and adherence to chronic medication, screening for malnutrition, TB, gender-based violence, and making referrals to health and social services. Registration of a household includes questions about coughing, HIV testing, pregnancy, recent births, children under five, chronic medication, and need for family planning, home-based care, social grants. In addition, in Sedibeng the CHW deliver chronic medication to elderly, or disabled, patients.

We categorized the CHW teams into the three types and selected two teams of each type (Table 1). We drew on the advice of the district managers, to select each pair of teams to maximise the similarity in the community served by the two teams.

Table 1. Configurations of CHW teams

Model	Supervisor	Based in	Site number
1	Professional & Enrolled Nurse	Clinic	1<br>2
2	Professional & Enrolled Nurse	Health post	3<br>4
3	Enrolled Nurse only	Clinic	5<br>6

<u>Data collection</u>

In each site we conducted a household survey, focus group discussions (FGD) with the CHW, observations of CHWs at work, and interviews with CHWs' clients, CHW supervisors, facility managers and the community representatives from Sept 2016 to Feb 2017 (Table 2).

Table 2: Data collection method, participants and data collected

Component		Participants	Total number in 6 sites	Data collected
Quantitative	Household Survey	Randomly selected households in the catchment area (220 per site)	1227 household interviews	• Socio-economic status of household, demographic profile, access to care, and types of need for care;<br>• Whether a CHW had visited in the past month/year (coverage);<br>• Health messages recalled by the HH (as a proxy for quality of care)
	Costs	Set up and recurrent costs per site	-	Set up and recurrent costs
Qualitative	FDG	CHW teams	12 FGDs (76 participants)	• Types of activities, challenges of the programme<br>• Self-administered questionnaire: age, years of training and service,

For peer review only

For peer review only - http://bmjopen.bmj.com/site/about/guidelines.xhtml

	Observations	CHWs and supervisors while conducting their daily work	126 days of observation	• Types of activities carried out; types of clients encountered; • Supervision activities and interaction with clinic staff;
	In-depth Interviews	Supervisors, facility managers, clinic staff, community key informants	43 key informant interviews	Perceptions of what the programme entailed, how it ran, its successes and challenges
		Follow-up interviews with CHW clients who were referred to the clinic during observations	74 household interviews	Whether client took referral action, and outcome client's perception of the service

<u>Household survey</u>

We used stratified sampling (based on area and housing type[13-15]) to select 220 households per site. Fieldworkers used a random walk and a specified skip pattern in a designated area to select households. The household was approached and the member who knew most about the health of other members, was invited to participate in the survey. Their responses were recorded on an electronic device. This allowed any irregularities to be identified and resolved as the survey progressed. We collected data on coverage (whether a household had been visited in the past year or in the past month), the need for care (number and types of health conditions), and the quality of care provided (the number of health messages recalled for visits in the last month). Health messages are pre-defined advice concerning each condition that the CHW was meant to provide, when relevant. Descriptive statistics were generated for all variables of interest and compared across sites. A logistic regression was conducted to better understand who was receiving CHW services. We included the following variables: model type, ratio of households per CHW pair, whether the household has a person over 60, a child under 5, number of health care needs, distance from the facility, and dwelling type (as a proxy for SES). We first conducted an unadjusted analysis to determine whether any of the variables were significantly associated ($p < 0.2$) with receiving a CHW visit. All significant explanatory predictors were included in the model, with those that were not significant ($p > 0.05$) being removed one by one, with the least significant being removed first. The analysis took into account the effect of clustering due to sampling and levels of strata by using a robust standard-error estimates for stratified sampling.

<u>Qualitative data collection</u>

Trained fieldworkers were given an orientation to community-based health care by an experienced primary health care nurse. Interview guides and observation templates were revised after internal piloting and feedback from fieldworkers. Respondents, except for CHW, were chosen purposively.

Focus group discussions

All CHW at work on the day of the focus group discussion were included. A brief survey captured key demographic and career history of the CHW. The topics discussed included descriptions of the types of activities carried out by CHW and the challenges they faced.

Observation of CHW

The selection of CHW to be observed was done randomly (drawing names out of a hat) on the first morning of a 4-day observation period. The fieldworkers observed the CHW with or without supervisor at work and took detailed notes in a template.

Interviews

Interviews were conducted with the facility manager, clinic staff, and CHW supervisors to discuss the typical activities of the CHW, how the programme ran, and its successes and challenges. If a client was given a referral by a CHW, the fieldworkers asked to conduct an interview with the client

For peer review only

For peer review only - http://bmjopen.bmj.com/site/about/guidelines.xhtml

in a month's time. This subsequent interview provided information on follow up actions taken by the client.

We drew together qualitative data to develop an explanatory description of how each team functioned, and then drew comparisons across teams. Firstly, we extracted data for each site into one document, either summarizing events, or useful quotations as raw data. (Team members extracted data from same sources, compared extracted data and modified extraction strategies until we were confident about inter-extractor reliability.) We presented a brief summary of each site in a one-day workshop, and through discussion identified common and divergent themes across the sites (such as weekly and daily pattern of activities, resources, record keeping, patient referrals to the clinic, engagement with clinic staff, relationship between CHW and supervisors, relationship with patients). We then collated data under these themes, producing a summary for each site (see example in appendix). This allowed us to further compare sites, to see how variation in supervision and integration with the health system influenced the functioning of the teams.

In addition, we counted the observed visits by purpose (medication delivery, follow up visit, registration), which questions were asked during registration, and, from the interviews with clients, whether the referral action advised by the CHW was taken and reasons why not. (Registration required the completion of a government form identifying nine health conditions/needs.)

Costing

We only included the costs to the health system, not household costs, or those incurred by CHWs such as airtime or transport. We included both set up costs (health post infrastructure and equipment, CHW kits, and training), and recurrent costs (staff costs, health post and equipment maintenance, CHW kit replenishment, and overheads). Staff costs included CHWs, supervisors and a share of the responsible district manager. Costs and the estimated life span of infrastructure and equipment were collected from the district management team. PHC expenditure per capita for the district was extracted from the Health System Trust District Barometer 2015. All costs are expressed in 2016 Rands after applying adjustment for inflation if required.

All items with an expected life over one year were treated as set-up costs and annualized by expected length of life, using straight depreciation. Costs were derived for each site, based on the particular site's staffing profile. Costs for the district CHW programme were calculated as expenditure per capita. This amount is compared to the per capita PHC expenditure to determine the share of PHC expenditure represented by the CHW programme.

Integrating the qualitative and quantitative evidence

We compared the data on coverage and quality of care from the survey with the qualitative evidence, in order to examine how the different models functioned, and why. We then combined these judgements with the costs to make an assessment about the value for money of the different models.

Ethics

The study was approved by the University of the Witwatersrand's Human Research Ethics Committee (Medical) (M160354), the Gauteng Provincial Health Research Committee, and the Biomedical and Scientific Research Ethics Committee (BSREC) at University of Warwick (REGO-2016-1825). Written consent was obtained from all participants. Verbal consent was obtained from household members for a fieldworker to enter the household to observe the CHW at work. Written consent was obtained from household members when subsequent interviewers were conducted.

Patient and public involvement

CHW clients and the public were not involved in the design, conduct, reporting or dissemination plans; however, facility, district and provincial managers were involved. We have conducted feedback sessions with CHW teams, facility, district and provincial managers.

For peer review only - http://bmjopen.bmj.com/site/about/guidelines.xhtml

RESULTS

The clinics, health posts and the CHW teams

The clinic in Site 1 was a formal, relatively modern building (Table 3). The clinic in Site 2 was prefabricated wooden structures with limited space, so the CHW had to meet outside. The health post in Site 3 (a repurposed transport container) was located relatively close to its 'mother' clinic. In contrast the health post in Site 4 was located in a community without a clinic. As a result, at this site, the PN supervisor spent half of her time consulting with patients, rather than supervising the CHW. Sites 5 and 6 were both formal clinics in rural settings. Site 6 had sufficient space for CHW to their meetings inside; Site 5 did not and CHW met outside.

The EN supervisors (other than in Site 1) were younger and had less work experience than the CHW. There were no PN supervisors at the two rural sites (sites 5 & 6); the district manager reported it was hard to recruit PNs to work in the rural clinics, because of their location. The size of the teams ranged from 9-20 CHW. More supervision time per CHW was available to the teams in sites 1 and 2, and to a lesser extent Site 3. Using the South African census, we estimated the number of households per pair of CHW varied from 120-400 (Table 3).

The average age of the teams varied between 33-41 years, and the percentage who had completed their high school education ranged from 25% to 63%. Length of time working as CHW ranged from 5-10 years. The majority of CHW had completed Phase 1 WBOT training (90-100%), except for the two rural sites (sites 5 &6) that were located over an hour's drive from the training facility.

The CHWs often worked without necessary resources (equipment, stationery, uniform, or funds for transport or mobile communication). The CHWs were meant to have a bag of equipment, which included a blood pressure machine, a glucometer and testing strips, a weighing scale, mid-upper arm circumference tape for assessing malnutrition in children, bandages, gloves, mask, forms, raincoat and water bottle. At the start of the fieldwork, many CHW were without bags. Equipment bags were delivered during the data collection but were not regularly re-stocked. In one team the CHW had not received training on how to use the manual blood pressure machines provided. The notebook and pen that CHW used to record daily activities had to be purchased out of their stipend, and so many of them did not have these items during our observations. They used pieces of papers to record the visits. On occasions, CHWs did not have the household registration form because the photocopier was broken. Two of the six teams did not have sufficient space to complete paperwork and to store their files; often files were kept at home.

The CHW were not formally employed by the government. They were an outsourced workforce and paid a minimal stipend for 6 hours work per day. CHWs struggled to contact the private payroll company responsible for monitoring attendance and paying stipends. An electronic system, requiring CHWs to clock in/out at the clinic, limited their ability to reach outlying areas. Two months prior to the start of our fieldwork CHW had been on strike over their conditions of employment. During the fieldwork the CHW were paid (2500 RSA), below the minimum wage (R3500 per month), however, by the end of 2018, it was increased to the minimum wage.

Household characteristics, need for, and access to, care

The proportion of households who agreed to participate in the survey ranged from 85-97% across the sites. Site 1 contained mostly formal dwellings, all of which had access to piped water inside their house (Table 3). In contrast, Site 4 was predominately an informal settlement, with only 3.8% of households having piped water inside the house; in Site 5, half of the dwellings were informal. Across the six sites, access to the internet ranged from 25 – 42% of households, with 31% of households having access in the informal settlement (Site 4). The age distribution of the population is similar with the informal settlements (Sites 4 & 5) having fewer children under 5, and the more

For peer review only - http://bmjopen.bmj.com/site/about/guidelines.xhtml

formal settlement (Site 1) having double the number of people aged 60 and over. The need for care was greatest in the informal settlements; in Site 5, 19% of households reported 3 different conditions or need for care due to high levels of HIV and TB. The formal settlement (Site 1) had higher levels of hypertension and diabetes due to its elderly population.

Household coverage

Across the sites 10-20% of households had been visited in the last year, with 5-12% visited in the last month (Table 4). Having only an EN supervisor was associated with a higher level of coverage. Those households with a person aged 60 and above were also almost twice as likely to receive a visit in the past year (due to the delivery of medication to elderly patients). However, other characteristics that we would expect to increase the likelihood of a visit, such as a child under 5, the number of health care needs, and dwelling type (a proxy for socio-economic status), did not. CHW were not more likely to visit households closer to the clinic or health post. Combining the household survey data with the estimated number of households in each catchment area, we estimated that a pair of CHW on average visited between 1-2 households per day. However, during the observations, the CHW visited on average of 3-5 households per CHW pair per day, the increase effort probably due to being observed.

Type, and quality, of care delivered

The purpose of household visits varied. CHW delivered medication in nearly half of observed visits (47%), followed up with a patient (38%) or registered a household not visited before (15%). None of the observed household registrations were fully completed, with CHWs completing on average 4-5 of the 9 questions (Table 5). The three most frequently asked about health conditions / needs were: a children under five, a person taking daily medication and a birth in the last 6 weeks (in >70% of registrations). The need for HIV testing and family planning was established in at least half of registration visits. Coughing, the need for social grants and pregnancy status were the least discussed. In a third of visits an additional health need was established, as CHWs asked about other health issues, although this varied across sites (72.5% in Site 4 and 18.2% in Site 3).

The survey respondents in sites 1 and 2 (with PN and EN supervisors) recalled a quarter of the relevant messages for the conditions discussed during the visit in the last month (Table 5). However, respondents in sites 4 (health post team in a community without access to a clinic) and 6 (one of the EN-only sites) recalled only 11% and 13% of the relevant messages.

Half of the clients sought care at the clinic when referred by the CHW. The most common reason given for no action was lack of funds or transport. In Site 6 only 39% of referrals were acted-on. Key community informants reported complaints had been made about staff attitudes at the clinic, which may have influenced whether the patient took action.

Supervision practices and support from the health system

We observed experienced supervisors using job training, supervised household visits, and debriefing sessions (during which problems were discussed) to train, motivate and monitor CHW, and improve the quality of their work. In one site, the PN gave brief education sessions about common health conditions in the community before the CHW left the clinic in the morning, "*I cover hypertension, diabetes, HIV, the children's Road to Health card, TB, health hazards, prostate and cervical cancer screening. It is things they deal with in the community.*" (PN, interview, Site 1). One health post-based PN/EN team (Site 3) held daily debriefing sessions, at which CHWs described the households visited, problems encountered, and actions taken. The sessions strengthened CHWs' knowledge base and problem-solving abilities, and provided a platform for collective supervision, enabling the CHWs to learn from each other's experience. Those teams that had no place to meet inside the facility tended not to discuss serious matters for reasons of confidentiality. *"Sometimes we stand outside to hold meetings… patients and the security personnel will be listening to what we are discussing." (CHW-FGD, Site 5)*

For peer review only - http://bmjopen.bmj.com/site/about/guidelines.xhtml

ENs routinely accompanied different pairs of CHWs on home visits several days a week. One EN (who worked as part of an EN/PN pair) demonstrated sensitivity in correcting CHWs' practice. *"When I can see this one didn't do it right, I keep quiet in the house, but immediately after we step outside as we are walking, I do on-the-spot training" (EN, interview, Site 2)*. Despite one EN (without a PN supervisor) spending 4 days a week out in the community, the CHWs did not acknowledge her as a supervisor, *"When we get there she's doing the same job that I normally do when I am alone." (CHW-FGD, Site 5)*

Job satisfaction, professional confidence and motivation were more evident in teams with a PN supervisor. One CHW commented that since the PN arrived: *"we started to feel that we are now CHWs and human beings." (CHW-FGD, Site 1)* When the CHWs were on strike over their conditions of employment, some of the CHWs at the clinic-based PN/EN Site (Site 2) tried to carry on, not wanting to let their patients down or damage their relationship with supervisors and the facility. Their supervisor interpreted their actions: *"they see that you, as their leader, are supporting them and trying to understand where they come from, especially with this employer thing being mixed up. That is why I am saying, hence, they refused to go on strike today." (EN, interview, Site 2)*

However, in the absence of supportive supervision in EN only sites, insufficient training constrained CHWs' ability to assist patients: '*since we work under WBOT we have not been trained,*" *(FGD Site 6).* Based an hour's drive from the district office, the rural sites rarely received training. With their frustrations over their unresolved working conditions, which were not acknowledged by their immediate supervisors, the CHW were demotivated: "*the way the contractor is operating, they make us to be lazy, because since we signed a contract with them, we have never seen them or had a meeting with them to tell us about their rules*" *(FGD Site 6).* Field notes also showed that many CHW did not make full use of their time due to a lack of motivation and ineffective time-task management. Some CHWs decided to work fewer hours a day as a passive protest.

In contrast, in the clinic-based teams, with the support of PN supervisors, and the resulting growth in skills and confidence, CHWs had been able to establish better relationships with clinic staff. In turn, clinic staff, seeing the CHW's greater motivation and effort, were more likely to treat the CHWs with respect. Communication and coordination was better. Working together, the facility manager and PN were able to confirm whether, once defaulters had been traced by CHWs, they returned to the clinic. *"We engage with facility manager, she helps with problems that we have... we include her in everything" (EN, interview Site 2)*. Clinic staff willingly accepted referrals from CHWs, and included the CHWs in training sessions: *"Sister X is not (in the CHW team), but she is very helpful. She attended severe malnutrition training and she called all the CHWs and did a presentation for everybody*." *(EN, interview, Site 2).* As a result the contribution of CHWs was highly valued: *"If they (CHWs) were not there, it means you will be over worked, the clinic will always be full, to be honest. There is good communication between us." (Facility Manager, interview, Site 1)*. However, even with a senior supervisor, if the team were located in a health post at a distance from the main clinic (*Site 4*), the CHW struggled to engage with clinic staff, and to ensure patients accessed to care at the clinic. (For a full description of the supervisory practices, please refer to our companion paper.[16])

<u>Value for money</u>

Across the district as a whole the annual expenditure on the programme was R44 million (2016 Rands), at an average cost per CHW of R52,000 (2016 Rands). The cost per capita (for the district population without private health insurance) was R47. District expenditure per capita on primary health care (including community health centres and clinics, but not district hospitals) was R1,200. We therefore estimated that WBOT expenditure in the district was only 3.9% of PHC expenditure.

For peer review only - http://bmjopen.bmj.com/site/about/guidelines.xhtml

The costs were higher in the models with: a) start-up costs of the health post; b) more supervisors, and c) a smaller number of CHW per supervisor (Table 5). The EN-only model in the clinic (Sites 5 & 6) was the cheapest; although this model achieved higher coverage, the observation data suggests the care provided was poorer. The health post at some distance from the clinic (Site 4) was mid-range cost but as the nurses were trying to provide a basic clinic service, the CHW programme suffered. The health post close to the clinic was expensive because there were a small number of CHW, but the quality of care was good. The PN & EN model (Site 1&2) in the clinic represented the best value for money as the team was well integrated into the health system, even if there was insufficient space in Site 2.

For peer review only

For peer review only - http://bmjopen.bmj.com/site/about/guidelines.xhtml

TABLE 3: Description of catchment area, households and members surveyed, and their need for care		PN & EN BASED IN CLINIC				PN & EN BASED IN HEALTH POST				EN ONLY BASED IN CLINIC			
		Site 1		Site 2		Site 3		Site 4		Site 5		Site 6	
**2016 DEPARTMENT OF HEALTH DISTRICT DATA**													
**Catchment area**	Population in CHW catchment area (N)	23,877		6,749		10,984		5,000		5,838		16,610	
	Household per site (N)	6,453		2,177		3,328		1,429		1824		4,746	
	No of people per household (Mean)	3.7		3.1		3.3		3.5		3.2		3.5	
**HOUSEHOLDS SURVEYED (n)**		**187**		**186**		**224**		**213**		**209**		**206**	
**SES, access to, and need for, care**	Proportion of households that were informal dwellings (%, n)	0.0	0	25.8	48	13.5	30	96.2	205	43.1	90	23.7	47
	Access to piped water indoors (%, n)	54.6	102	39	73	53.6	120	3.8	8	44.0	92	64.6	133
	Access to internet (yes) (%, n)	42.3	79	25.8	48	33.9	76	31.0	66	25.4	53	32.5	67
	Walk to the clinic (yes) (%, n)	77.4	144	63.4	129	88.8	199	74.7	159	89.0	186	87.3	179
	Distance from house to clinic / health post (km) (median, IQR)	0.7	0.5-1.0	1.7	1.5-2.2	0.4	0.2-0.7	0.4	0.3-0.6	2.4	2.0-2.9	0.6	0.4-1.3
	3 or more health conditions or needs (%, n)	18.2	34	12.4	23	11.6	26	16.4	35	19.6	41	14.1	29
**INDIVIDUALS IN HOUSEHOLDS SURVEYED (n)**		**692**		**574**		**747**		**755**		**679**		**722**	
**Age**	0-4 (%, n)	6.5	45	7.8	45	7.9	59	3.8	29	4.3	29	7.9	57
	5-18 (%, n)	19.4	134	26.7	153	26.9	201	26.8	202	25.9	176	28.5	206
	19-59 (%, n)	58.7	406	59.6	342	58.5	437	61.6	465	63.0	428	56.1	405
	60+ (%, n)	15.5	107	5.9	34	6.7	50	7.8	59	6.8	46	7.5	54
**CLINICS, HEALTH POSTS AND CHW TEAMS**													
Description of clinic or health post and availability of space		Modern clinic building		Prefabricated clinic building		Transport container, close to 'mother' clinic		Prefabricated building some distance from 'mother' clinic		Modern clinic building in rural setting		Modern clinic building in rural setting	
Does the team have a room inside the facility to use for meetings?		Yes		No		Yes		Yes		No		Yes	
Number of CHW per team		16		17		9		12		14		20	
No of households per CHW pair		403		128		396		119		130		237	
Proportion of CHW who have finished high school education		27.3%		35.7%		50%		63.6%		25.0%		33.3%	
Proportion of who have passed Phase 2 training		90.9%		24.4%		100%		45.5%		0%		0%	
Number of professional nurse supervisors		1		1		1		1		0		0	
Number of enrolled (junior) nurses as supervisors		2		2		1		1		1		1	

Note: Distance = direct distance between GPs co-ordinates

For peer review only

Table 4: Household coverage and quality of care

		PN & EN BASED IN CLINIC				PN & EN BASED IN HEALTH POST				EN ONLY BASED IN CLINIC			
		Site 1		Site 2		Site 3		Site 4		Site 5		Site 6	
Coverage	**All households SURVEYED (n)**	**187**		**186**		**224**		**213**		**209**		**206**	
	Households visited by CHW in the last year (%, n)	10.2	19	11.8	22	15.6	35	16.0	34	19.6	41	19.9	41
	Households visited by CHW in the last month (%, n)	5.3	10	7.5	14	8.9	20	6.6	14	12.4	26	5.8	12
Purpose of visit, whether registration completed or additional need	**Household visits OBSERVED (n)**	**81**		**87**		**77**		**40**		**89**		**128**	
	Household Registration (%, n)	27.1	22	6.8	6	-		72.5	29	-		16.4	21
	Medicine delivery (%, n)	66.7	54	48.3	42	59.7	46	20.0	8	49.4	44	33.6	43
	Follow up (e.g. missed clinic appt, hospital discharge) (%, n)	6.2	5	44.8	39	42.8	33	7.5	3	50.6	45	51.6	66
	Average number of registration questions asked (%, n)	53.5	4.8/9	55.5	5/9	-		50.2	4.5/9	-		43.9	4/9
	Visits whereby CHW found a need (%, n)	60.0	49	27.5	24	18.2	14	72.5	29	19.1	17	35.9	46
Advice recalled	**All households SURVEYED (n)**	**187**		**186**		**224**		**213**		**209**		**206**	
	Proportion of relevant health messages recalled (ratio, SE)	0.27	0.03	0.26	0.03	0.18	0.03	0.13	0.04	0.20	0.02	0.13	0.07
	Proportion of messages recalled for hypertension (ratio, SE)	0.28	0.02	0.27	0.03	0.21	0.04	0.12	0.05	0.24	0.02	0.19	0.09
Referrals acted upon	**Total individual interactions during OBSERVED HH visits (n)**	**127**		**128**		**112**		**79**		**136**		**165**	
	Number of referrals recorded (%, n)	12.6	16	13.3	17	17.0	19	15.2	12	19.1	26	10.3	17
	Number of referred individuals interviewed (%, n)	-	14	-	13	-	15	-	6	-	16	-	13
	Patients who took referral action (%, n)	57	8	54	7	53	8	50	3	69	11	39	5

For peer review only

Table 5: Annualized costs of the different models

RANDS 2016	PN & EN BASED IN CLINIC		PN & EN BASED IN HEALTH POST		EN ONLY BASED IN CLINIC	
	Site 1	Site 2	Site 3	Site 4	Site 5	Site 6
Number of PN supervisors	1	1	1	1	0	0
Number of EN supervisors	2	2	1	1	1	1
Total costs per team	1,047,953	1,083,809	938,344	910,240	694,429	909,564
Number of CHW	16	17	9	12	14	20
Cost per CHW	65,497	63,753	104,260	73,853	49,602	45,478
Explanation for variation in costs	Two ENs in each of these sites, so total costs are higher, but the teams are larger so supervision is needed		Fewer CHW per supervisor so more expensive, as well as the costs of the health post	Larger team so cheaper than Site 3, but one supervisor actually consulting patients	With a fewer supervisor and larger teams, the costs were lower	
Quality of care	High quality		High quality	Poorer quality because less supervision and significant distance to facility	Poor quality, because insufficient supervision	
Value for money	Good value: good supervision, and well integrated into the health system, even in clinic where there is not enough space for the CHW		High quality model, but intensive because few CHW, and so expensive.	In mid-range in terms of expense but trying to provide basic clinic services as well, so quality suffers	Poor value: Cheaper but poor quality, ineffective care	

For peer review only

DISCUSSION

In summary, household coverage was limited in all the sites (10-20%). The low proportion of registration visits suggests that the CHW were revisiting households they know (driven by the requests by the clinic to deliver medication or trace patients who had stopped attending the clinic) rather than taking on the more difficult task of approaching an unknown household, and so limiting the number of households they were responsible for. Insufficient training and resources demotivated the CHW and generated resentment. Our study suggests that the CHW could achieve a greater number of visits per day, and that more effort could be made towards targeting households in greater need (e.g. those with young children). An increase to four visits per CHW pair per day would achieve coverage ranging from 30-90%, suggesting that some teams need more CHW, as well as better supervision. Across the 6 sites, assuming that CHW work in pairs for security, they spend 4 days a week visiting households[1], make 4 household visits a day (1 household registration visit and 3 follow up visits per day), a pair of CHW could care for approximately 220 households.

With the respect to quality of care, from the observations, we learnt CHW did not ask all the registration questions; without knowing the full range of health and social needs of the households, they are unlikely to provide the necessary care. However, the CHW identified additional need(s) (beyond the purpose of the visit) in a third of households. From the household survey, we learnt household respondents remembered between a fifth to a quarter of the health messages, and half of patients took the referral action recommended by the CHW.

With respect to supervision and location, where there was better, more senior supervision, household members were more likely to recall the advice and messages given; where there is less supervision and less training, the CHW are visiting more households, but providing poorer quality care. Teams based in a clinic, with a senior supervisor, are more likely to be better integrated into the health system, and so able to provide a higher quality service. Without senior supervision the EN supervised teams, although located in a clinic, were not integrated into the system as they didn't receive the necessary training or on-going support, and they struggled to negotiate effective working relationships with other facility staff members. In the health post that served a community without a clinic, nurse supervisors had to consult directly with patients and had less time to supervise the CHWs, and engagement with the clinic was difficult due to the distance.

Expenditure on the CHW programme is only 3.9% of expenditure on non-hospital primary health care. Greater investment in more capable supervision, and where necessary, a greater number of CHW, would increase coverage, and improve the quality of the service. Box 1 sets out our recommendations from this phase of the study. Given the shortage of professional nurses, scaling up the PN & EN model is not feasible, so cost-effective ways need to be found to train and provide on-going to support to the EN supervisors in order to improve the quality of their supervision. In the second phase of this study, we are evaluating at an intervention to provide mentorship to the EN supervisors in sites 5 &6.

Limitations

Due to the very low number of households reporting a CHW visit in the past month, analyses of specific services were not possible. As a result, we have relied on the observation of household registration visits to assess the comprehensiveness of the service provided. The number of messages received during the CHW's visit in the last month, reported by the household member, depended on their recall. During observations, the work effort by the CHW increased due to the presence of fieldworker; we observed the same CHW over 4 days in order to reduce this effect. However, we were also able to estimate the likely number of visits made per day from the household survey

[1] Other days will be used for campaigns, leave or compiling statistics.

For peer review only

results. We weren't able to do a cost-benefit analysis, and have instead compared the costs to overall primary health care expenditure to assess affordability, and value for money.

The study's strength is the wide range of data collection methods that enabled triangulation across the different data sources, including the observation of CHW activity rather than self-reported data. The six sites were typical of the Sedibeng District, and while it is not possible to generalise with any certainty, the sites included both rural and peri-urban communities typical of much of the country.

Sufficient numbers of CHW to achieve adequate coverage is clearly important, however, the optimal number of households per CHW depends on her/his role, as well as geographic characteristics of the area.[7, 17] Most evaluations of CHW programmes are focused either on a single condition, or a cluster of conditions (e.g. maternal and child health); there is limited research assessing the performance of CHW in more comprehensive programmes. Where delivery of care is focused on maternal and child health, CHWs have been found to struggle with providing comprehensive care, with gaps in the information provided by the CHW, due to insufficient supervision and support from the associated health service [18, 19] In Rwanda, the addition of family planning to CHW's wide range of promotive and preventive tasks didn't place an undue burden on the CHW, although it was noted there were insufficient CHW to meet the family planning needs of their catchment area.[20] In response to the HIV epidemic, studies examining the expansion of CHW programmes to include ARV treatment found sustainable and consistent funding to be the key barrier.[21]

Effective linkages with the formal health system are key, including being perceived by other health workers as an integral and essential member of the health team, and as the foundation of the health system.[7] Several recent studies have focused on the degree of integration of CHW with the health system, identifying barriers such as resistance from other health workers, discrimination against CHW based on social, gender and economic status, ineffective incentives, inadequate supplies and infrastructure [22-24]. An evidence review found that in large scale programmes, supervision is almost always weak, with those responsible for supervision frequently having other responsibilities (such as patient care at a peripheral facility), and they often have no specific training in supervision of CHW.[25] Weak management and organizational structures lead to poor quality work, low morale and absenteeism.[26] The transition to comprehensive care in the Western Cape Province in South Africa, was hampered by weak support systems (supervision, monitoring, financing, training) as well as insufficient sub- district capacity for planning and management.[10]

There is little research on both household coverage and the quality of care provided; this is important as there is a potential trade-off between the two. In addition, there is little on evidence the synergistic effect of senior supervision, which may be important in enabling integration with the health system, and improving both coverage and quality of care. Given the shortage of human resources of health in low and middle income countries, innovative strategies to strengthen supervision strategies are required.[7, 27]

For peer review only

Box 1: Recommendations for an effective CHW programme

1. Dedicated, experienced supervision (both to build CHWs', and their supervisor skills, and to facilitate collaboration with the closest facility) is essential otherwise the investment in CHW programme is unlikely to achieve the household coverage or quality of care possible.
2. Where there are insufficient numbers of CHW, the programme needs to comprise a larger proportion of the PHC budget, in order to substantially increase the coverage of the programme;
3. A room inside the clinic to meet and compile reports is a necessary but not sufficient for the integration between clinic and the CHW team. Where physically not possible, an additional structure should be build next to facility
4. Health posts in locations without a formal clinic to serve the community should be converted into formal clinics, or at least provided with sufficient resources and infra-structure so that they can operate as a full clinic.

CONCLUSION

The CHW provided their service to less than a fifth of households in the catchment communities. The service the CHW provided to the households they visited was limited. Where skilled senior supervision was available, the CHW were able to provide a service that covered a broader range of health needs. Support from the local facility for the CHW team is key to providing a quality service, and a senior supervisor can help to facilitate this collaboration. Greater investment in numbers of CHW, supervisors, training and equipment is required for the potential benefits of the programme to be delivered.

Data Availability
Quantitative data can be made available on request. Given the difficult of anonymizing qualitative, we will work with any researchers wishing to further analyse the qualitative data.

Acknowledgements

We would like to thank all of the study participants, as well as district and provincial management for their support and engagement with the study

Funding Acknowledgements

The study was funded by Medical Research Council UK, DFID, ESRC, and Wellcome Trust under the Joint Health Systems Research Initiative

Author Contributions
JG, MT, FG conceptualised the study and raising the funding.
JG lead the drafting of the manuscript.
JG, JDK, OB, HM, were responsible for overseeing data collection
ED was responsible for costing both data collection and analysis
MM, JL, JDK, OB were responsible for statistical analysis
JG, FG, HM, YT, TR, NN, JAW were responsible for qualitative analysis
JG, FG, HM, YT, TR, NN, JAW, MT, OB contributed to the drafting the manuscript
All authors have read the final draft.

Author Characteristics
JG is Professor of Health Policy and Systems at Centre for Health Policy
JdK during the study was a researcher / project manager at the Centre for Health Policy

For peer review only

OB is researcher / project manager at the Centre for Health Policy
MM during the study was a bio-statistican at School of Public Health
MK during the study was a post-doctoral fellow at Centre for Health Policy
HM & TR are PhD fellows at Centre for Health Policy
NN is a senior researcher and Deputy Director at the Centre for Health Policy
JL is Professor of Biostatistics at School of Public Health
MT is Emeritus Professor at Warwick Medical School
ED during the study was a health economist at the South African Medical Research Council
JAW is a researcher at Warwick Medical School
FG is Professor of Medicine and Society at Warwick Medical School
None of the authors had any prior interaction with the respondents.

Data

Quantitative data can be made available on request. Given the difficult of anonymizing qualitative, we will work with any researchers wishing to further analyse the qualitative data.

Declaration of Conflicting Interests

Nothing to declare.

References

1. Zulliger, R., M. Moshabela, and H. Schneider, *"She is my teacher and if it was not for her I would be dead": Exploration of rural South African community health workers' information, education and communication activities.* Aids Care-Psychological and Socio-Medical Aspects of Aids/Hiv, 2014. **26**(5): p. 626-632.
2. Walley, J., et al., *Alma-Ata: Rebirth and revision 8 - Primary health care: making Alma-Ata a reality.* Lancet, 2008. **372**(9642): p. 1001-1007.
3. Christopher, J.B., et al., *Thirty years after Alma-Ata: a systematic review of the impact of community health workers delivering curative interventions against malaria, pneumonia and diarrhoea on child mortality and morbidity in sub-Saharan Africa.* Human Resources for Health, 2011. **9**.
4. Glenton, C., et al., *Can lay health workers increase the uptake of childhood immunisation? Systematic review and typology.* Tropical Medicine and International Health, 2011. **16**(9): p. 1044-1053.
5. Pallas, S.W., et al., *Community Health Workers in Low- and Middle-Income Countries: What Do We Know About Scaling Up and Sustainability?* American Journal of Public Health, 2013. **103**(7): p. E74-E82.
6. Tulenko, K., et al., *Community health workers for universal health-care coverage: from fragmentation to synergy.* Bulletin of the World Health Organization, 2013. **91**(11): p. 847-852.
7. Perry, H. and R. Zulliger, *HOW EFFECTIVE ARE COMMUNITY HEALTH WORKERS? An Overview of Current Evidence with Recommendations for Strengthening Community Health Worker Programs to Accelerate Progress in Achieving the Health-related Millennium Development Goals*. 2012, John Hopkins Bloomberg School of Public Health: Baltimore.
8. World Health Organization, *Working together for Health*, in *The World Health Report*. 2006, World Health Organization: Geneva.
9. SADoH. *Provincial guidelines for the implementation of the three streams of PHC Re-engineering*. 2011 [cited 2017 23/January/2017]; Available from: http://www.rmchsa.org/wp-content/resources/resources_by_type/DistrictLevelResources/ProvincialGuidelines_PrimaryHealthCare3StreamsOverview2011.pdf.
10. Schneider, H., et al., *The challenges of reshaping disease specific and care oriented community based services towards comprehensive goals: a situation appraisal in the Western Cape Province, South Africa.* BMC health services research, 2015. **15**(1): p. 436.

11. National Department of Health, *Policy framework and strategy for ward-based primary health care outreach teams*. 2018, Republic of South Africa: Pretoria.
12. Gomm, R., M. Hammersley, and P. Foster, *Case study method: Key issues, key texts*. 2000, London: Sage Publications.
13. Ryu, E., et al., *Assessing health disparities in children using a modified housing-related socioeconomic status measure: a cross-sectional study.* BMJ Open, 2016. **6**(7): p. e011564.
14. Dunn, J.R., et al., *Gender differences in the relationship between housing, socioeconomic status, and self-reported health status.* Rev Environ Health, 2004. **19**(3-4): p. 177-95.
15. Juhn, Y.J., et al., *Development and initial testing of a new socioeconomic status measure based on housing data.* J Urban Health, 2011. **88**(5): p. 933-44.
16. Tseng, Y.H., et al., *Integrating community health workers into the formal health system to improve performance: a qualitative study on the role of on-site supervision in the South African programme.* BMJ Open, 2019. **9**(2): p. e022186.
17. Earth Institute, *One Million Community Health Workers: Task Force Report.* 2011, Columbia: New York.
18. Wilford, A., et al., *Exploring the care provided to mothers and children by community health workers in South Africa: missed opportunities to provide comprehensive care.* BMC public health, 2018. **18**(1): p. 171.
19. Haskins, J.L., et al., *Fragmentation of maternal, child and HIV services: A missed opportunity to provide comprehensive care.* African Journal of Primary Health Care & Family Medicine, 2016. **8**(1): p. 1240.
20. Chin-Quee, D., et al., *Balancing workload, motivation and job satisfaction in Rwanda: assessing the effect of adding family planning service provision to community health worker duties.* Reprod Health, 2016. **13**: p. 2.
21. Wringe, A., et al., *Delivering comprehensive home-based care programmes for HIV: a review of lessons learned and challenges ahead in the era of antiretroviral therapy.* Health Policy Plan, 2010. **25**(5): p. 352-62.
22. Zulu, J.M., et al., *Integrating national community-based health worker programmes into health systems: a systematic review identifying lessons learned from low-and middle-income countries.* BMC Public Health, 2014. **14**: p. 987.
23. Oxford Policy Management, *Lady Health Worker Programme: External Evaluation of the National Programme*. 2009, Family Planning and Primary Health Care- Systems Review;.
24. Uys, L.R., *The practice of community caregivers in a home-based HIV/AIDS project in South Africa.* J Clin Nurs, 2002. **11**(1): p. 99-108.
25. Berman, P. and L. Franco, *Formal Health System Support Activities and Community Health Worker Performance.* 2012, Global Health Evidence Summit: Community and Formal Health System Support for Enhanced Community Health Worker Performance.: Washington, DC.
26. Prasad, B. and V. Muraleedharan, *Community Health Workers: A Review of Concepts, Practice and Policy Concerns:*. 2007, International Consortium for Research on Equitable Health Systems (CREHS);.
27. Cometto, G., et al., *Health policy and system support to optimise community health worker programmes: an abridged WHO guideline.* Lancet Glob Health, 2018. **6**(12): p. e1397-e1404.

For peer review only

Case site summary

Site: XXX Author(s): JG Date:22 02 2017

**Model**	Clinic based team, although this is a substantial, but temporary structure and doesn't perform all standard clinic services (e.g. TB)
**Nurses** background/ experience, age, role, actual responsibilities	[background deleted for anonymity]<br><br>**PNO:** once CHW have gone into the field, and have explained several things to the FW, spends an hour seeing patients.. and then leaves (so no meeting after CHW return)<br><br>**PNO_NM:** interview with FW, patient consultations for 2 hours; after 11.20 she reads patient files / notebooks; She also see family planning patients; (FW-PN doesn't seem to have a lot of work to do)
	**Observation day 1:**<br>Creche campaign; EN is leading activities<br>**Observation day 2:**<br>Works in clinic as short-staffed; sorting patients and then doing child immunisations;<br>Comes out to check on CHW to allocate them to FW<br>HH1 CHW leads<br>HH2 CHW checks patient, but EN worried so asks questions about stomach<br>HH3: EN leads<br>HH4: EN asks about granny; in hospital; EN is shocked because here only 2 weeks ago, checks whether she has enough medication etc *(suggests that EN02 does go regularly to the field)*<br>HH5: jointly doing activities<br>HH6: CHW leads, didn't ask about child<br>HH7: Jointly leading<br>Return to clinic at about 12 noon<br>*FW comment: EN is very supportive of CHW*<br>**Observation day 3:**<br>HH1: on way to support group visited a lady who had given birth to twins- just checked on babies<br>Support group: EN sits at a distance, CHW know how to do their work; she only intervened when a BP was too high, and when difficult TB case arrives (HH interview: CHW bring sputum bottle, but patient fails to provide) ; not 12 noon so there is time to see a few households<br>HH2: not allocated to a CHW, but she checks on him because he is sick with TB; EN leads (as she hasn't been before); EN says they will come and do registration even though not in demarked area<br>HH3: car accident victim and HIV<br>HH4: EN quiet, CHW leads<br>Back on counsellors place by 1pm after 30 mins walk<br>**Jane:** *EN seems works hard, happy to go and find extra households, seems to know some households – evidence that goes into the field regularly*<br>**Observation day 4**<br>Discussion about working on Fridays they do stats – CHW said we don't; EN said that is only the last Friday of the month .. so today they are going into the field; Later EN says later .. eish we are working on a Friday .. this is slavery<br>HH1; came to give referral letter for AGAPe .. which is now closed for the year;<br>CHW says I will take you to the patient you didn't manage to see on Monday<br>HH2: patient discharged from hospital in pain; EN tried to help – offered to call an ambulance, but woman said no because nobody to go with her; suggested clinic on Monday – ok, daughter will take me; asked for daughter's number; can't get it out of the phone<br>HH3: checking on patient that they referred two days ago; she hadn't gone ; doesn't take her treatment; child without immunization; EN quite upset<br>HH3: healthy baby; EN happy ☺
**CHWs** – average details across team (Number, age, gender, years' experience, education)	14 CHWs in FGD, (17 reported to part of team) , all female [info deleted for anonymity]<br>Average age 39 (range25-49)<br>64% of have some secondary education;<br>29% have matric<br>7 years on average as a CHW (range 3-16)<br>Average number of households=262 (248-317)<br>25 mins walk from home to clinic (range 2-50)<br>18 mins walk from clinic to households (2-45)

For peer review only

	93% have phase 1; 21% have phase 2
Community profile (eventually draw in survey data)	**PN:** We started going from house to house, counting people during the registration.. according to where the CHW stayed.. initial plan was 250 households, but didn't work .. either 190 or 200 (if medical aid we skip it)<br><br>People report to us if somebody is sick, they will even break down the door<br>Area 1)– area is huge - shortage of CHW<br>3 areas within Area 1<br>Ext 4 and 5 informal settlements / shacks– 5 is too big<br>Ext 2 – RDP house<br>People are moving to Rdp houses, go back 3 days later and people have moved..<br>Lots of Zim patients, but language not a barrier<br>Informal settlement, lots of crime, didn't feel safe at beginning,, but not easy for CHW to be robbed because most of them are well known in community
Challenges? (Not general)	Storage is a problem – for files,<br>Safety .. too many break ins in the clinic<br>CHW need more training, and then more CHW will hopefully come later<br>**FDG**<br>Equipment **–** one CHW has take the BP machines that side .. so you call her to ask her to bring this side .. call from counsellor .. and we don't have machines<br>Behaviour of nurses – some patients refuse to come to the clinic because of the bad treatment. However we encourage them for the sake of their health to ignore the nurses attitude P11<br>Queuing – it irritates the patients .. so it gives us a problem when we try to encourage the patients to go to the clinic ..<br>Clocking in WBOT promised we would work around where we stay.. they promised a clock in Area 1 .. we have found a place for it .. it is tiring to have to come to Area 2 to clock in .. go back to Area 1 and then come back to the clinic .. it is a distance and it is hot or raining!<br>Stipend, job security, career development, smart purse<br>**FDG**: as above plus job safety, training, recognition and appreciation<br>We can't even go to the bank with this card, only to shoprite .. otherwise they charge us<br>**Obs:** currently leave is pause .. because smart purse says 13 days, contract says 21 days – hey have sent a letter to smart purse – EN really interested in solving problems .. but sometimes above her
Planning (of the day)	I have paired them, an intelligent one with a less intelligent one
Weekly pattern of activity (what do they do when?)<br>- overtime	Run support group for elderly patients on Wednesday<br><br>**FDG:**<br>We follow up on defaulters<br>Household registrations each day, - it is not possible to do registrations at one time of the year<br>Home-based care – we don't do much of this ..<br>we refer to social services<br>*Jane: not convinced there is a day for this, and a day for that .. rather that they do all of the activities when needed.*<br><br>**FDG:**<br>With Kgatelopele the first week I deliver medication, the second I will check if they are taking it properly by counting.. if it doesn't balance .. then I will ask her why .. then I will report to the clinic to the clinic that I am following up on this person .
Daily pattern of activity	6 hh per day (for two CHW) (average is 5)<br>13 mins average per household (average is 12)<br>10 mins average travelling between households (average is 14)<br>5 hours work per day (average is 4.40)<br><br>5% of activities are meeting with supervisors (not time)<br>20% of activities are HIV as condition; (TB 5.5%)<br>52.75% of activities are other chronic<br>5.49% as mother and child<br>53% of recipients are elderly<br><br>Between 8-9 am do training<br>**EN02**: training and gym until 9am.. e.g. with new machines I take two CHW and show them so that they know what to do in the field

For peer review only

	You can see a big difference between those who have attended the trainings..<br><br>On a Tuesday they are taking medication to patients, and from there to check on what ever<br>So if we have gone to 2 households, taking treatment, then I pick one house, any house that we haven't been to before ..and do a registration ..<br>Cos you find that their focus is on the patients that they know, the patients that they know<br>But with me I am always squeezing in just one house that has never been registered .. so I don't totally change their routine .. just one HH and you will find problems<br><br>8am clock in and wait outside until 9am when go to households (no mention of training)<br>CHW work in pairs must see 6 households 3, 3, lunch break is between 12-1<br>They don't just to go their patients, they also visit other households after a longer break when no new patient is identified. People in the area also inform the CHW when there is somebody who is sick;<br><br>**FGD:** 6 households per day each and households registrations is too much .. and we have to be back here by 13.30 .. our lunch is at 12 noon. We discuss issues we have with the nurse and pack medication to deliver (next day or on way home)?)<br>We write everything in our note book .. and then we note in our diary when we have to return to a household<br><br>**FDG:** planning seems to be more about which of your houses and which of mine<br>• Last time they told us we had to see 6 patients a day per CHW .. now they have changed it to 4<br>• If you are doing household registrations .. you can't do 6 per day p13<br>• Sometimes we do home-based care, if the patient is living on her own and she is on treatment . .if ask if she has eaten .. if she has't I cook for her;<br>• We refer them .. the provider needs 8rand<br>• Sometimes we teach the family how to bath them
Record keeping & Stats – how & when<br>- How notes/info are kept<br>- How they are consolidated<br>- Patient files<br>- Follow ups	Has improved since two ENs arrived<br>WE don't have email and our transport is irregular;<br>Now doing it once a week .. we used to do it once a month .. but then it was late<br>Two ENs are taking responsibility and they forward to the data capturer and then we come back and chat about it (Sister T and FM?)<br>they have notebooks that they write in .. not more than 5/6 households per notebook .. (*so does that mean that they have 40 notebooks?)* I read those books every time I get a chance<br>she does monthly stats, she checks individual stats, talks to CHW saying why do you say this, or why haven't you write x, gives training to CHW.<br>In training we were told it would be easier if we did it weekly so I said to CHW lets do it on Fridays .. lets not go ot households but do stats instead .. now we are doing it monthly because we are faster now<br>From CHW to EN to facility manager<br>*Jane: this is why they don't work on Fridays.*<br>we do stats daily .. at lunchtime we record evertying (some confusion about recording number of children)<br>**:** the OTL takes time to check them<br>I do them in my lunch hour, or at home, or when we are seeing my partner's patients
Referrals<br>- Forms/documentation<br>- Queueing<br>- Files	Patient meant to show form at door and then goes to see Sister T, who then completes the form and gives back to the carer (if another nurses see patient they also complete the form)<br>Report number of referrals in stats as well<br>**FDG**<br>"The referral form is causing trouble with the patients. The patients are supposed not to follow the queue, they should go straight to the PN but the patients are been shouted, told to go and follow the queue. Now the patients are fighting with us because we told them that with the referral they don't have to queue."p 15
Medication delivery	ENs can't come issue medication; so T does it . and if she is not here, they come to me with the vital signs data and the pill count, the condition of the patient, and the next clinic data. If the ENs are there the CHW will do this<br>**FDG:** when you count their medication, sometimes they start shouting at you . you try to tell them that I am trying to help you ..
Relationships Clinic – WBOT team	Other sisters will give medication out if necessary<br>CHW refer patients to specific sisters eg. For TB<br>We do in-service training .. Sister Th is doing EPS .. she is very helpful .. she is not WBOTS but she is very helpful - she is a staff nurse, but she has got more experience ... she attended severe malnutrition training ..

For peer review only

	she called all the CHW and patients .. she as doing a presentation for everybody .. so it is not only us doing that .. She is also helping when we need something There is support from the clinic.<br>We engage with the FM for everything, problems we have, reporting .. she is part of the community, we include her in everything<br>District coordinator – I have only seen her once, when she came with those two ladies from wits !<br>Training person at johann heynes – if I have a problem a message the OTL group, or even phone her .. she is giving support .<br>**FDG**<br>"At the clinic we are treated as if we are not part of the clinic, they talk to us as if we are children. They shout at us in front of the patient that you brought for help. We don't have support at all. Another thing, they don't want us to eat breakfast but they eat when they come to the clinic. We consult TB patients on empty stomach. I did raise that to the PN and she said we should eat at home. Then I asked why she is not also eating at home in the morning and see if she can do it. In the morning we prepare our children to go to school, others have husbands to take care of before we leave at 7:00am, and we have to walk. We don't have time to have breakfast at our houses in the morning. We only have breakfast and lunch together at 12:00pm. There is no support at all." P15<br>**FDG:**<br>"the sister in charge and our OTLs are treating us like.. I don't know .. as you can see we have no place to stay, no chairs, no table, nothing .. we came with the idea of starting the day with a prayer like in hospital.. but the sister in charge says we won't do such a thing here .. so we stopped because we are powerless' p14<br><br>**Any conflict with OTL or clinic staff**<br>"We are all going to talk neh .. add to what I say .. we have our sister in charge, but we have another one .. the CHWs know who I am talking abut .. this sister is controlling us more than our OTL" .. P21<br>"she even shouts at us in front of the patients. We are adults, mothers and we have our own houses and when we are here she treats us like children. She does not respect us." P21<br>"And sometimes we walk in, she shouts and says 'hey you is that a uniform, that is not the way we dress here.' Where are we going to get the new navy pant? That pant costs R190-R200.00. All that shouting she does it in front of everyone at the clinic. That is not okay and when you talk back to her, she will say that she is not our OTL and does report to us."<br>**Participant**: "We want her to be out of the WBOT team. We want to report to our OTL only. She is responsible for other nurses not us."<br><br>"My challenge as a CHW is that when I do home visit, some households do not have food to eat and the patient is on treatment. So I have to empathise with the patient, contribute something so that the patient can eat. When I come to the clinic and ask for this porridge, the clinic staffs tells me that I have to come with the patient so that they can see the patient. That is a big problem because the patient is sick and has no transport money to go to the clinic. So yah it hurts." P20
Supervision of CHWs (including training)	I read their books whenever I can; if they have difficult cases they bring them straight to me .. and I take the car and drive to see the issue ..<br>Most of the CHW are older than her.. they don't always listen to the EN. When they only have one household to visit they have all sorts of stories to get away. She reported this to the FM who said she would sort it out.<br>Two CHW communicate on behalf of the rest ..<br>They report to the EN when they have found a defaulter, they then revisit and then report<br>Weekly or bi-weekly she checks the activities of the CHW<br>FM told CHW not to come to work during strike<br><br>supervises 8 CHW, 5 have not been registered so they are using the book. If she knows CHW have much work to do, she leaves with them at 8 rather than 9<br>Different types of CHW – counsellor, peer educators, rest are CHW; one CHW is the leader whose job it is to forward complaints to her<br>If I have been on training I will ask how did it go with that patient, we have an whatspp group so we can communicate that weay .. if I am not around, they will send me a whatsapp and I will say do this or that.<br>I spend the whole day with them, .. and then I give them a lift home

For peer review only

	Since the strike they have not been the same .. some they understand, some they are aggressive … so I said if you need something, or clarity, sit as CHW, write a memo /list and let your head CHW delivery it .. according to the hierarchy – this really helps, otherwise we have too many complaining and nothing will be sorted out e.g. bags – why only 10 bags; so they wrote it down, I took it to my PN, and the facility manager who was able to sort it out; we discussed how they are going to use the equipment<br>*Jane – example of sorting things out*<br><br>It goes back to the in-service training … when I am compiling the stats I am able to see this one is struggling .. when I do supervision I can see this one didn't do it right … I keep quiet in the house,, but immediately we step outside .. as we are walking I do spot training<br><br>Both had stories of truancy (going home and coming back to clock out);<br>uses her car to drive them back to the facility if they need to clock out, or allows them to sign out tomorrow if they are working late<br>Sometimes they come back early because of the distance.. because it can be an hours walk … you can waste 2 hours a day walking backwards and forwards<br>**FDG** do you work overtime? If we do household registration, and we don't find anybody who can has the information, we go back in the afternoon or the weekend .. .. Sometimes we deliver medication in the afternoon.. sometimes we are do vital signs at 7.45<br>**FDG:** if some people are very sick we monitor them over the weekend<br>Our team leader is supportive .. if the patient wants to know something .. I ask her
Supervision of ENs	do induction and appraisals of EN; provide training – HIV (going on Monday) , TB (one has gone the other will go in 2017), EVP<br>if sister X is not around she goes to the FM and she provides a solution<br>They help out between 7.30 and 12.00 at the clinic when the clinic is full. When the clinic needs them they pick two CHW to stay and help the EN .. but they are free to say no<br>The facility manager makes sure we attend regular training to uplift ourselves She organizes workshops with CHW to train them on what she has learnt<br>Now I can lead campaigns – cos I understand everything about the campaign – so you see there really is development here..<br>My job is with WBOTs. 70% in the field, 30% in WBOT admin .. if I am not busy I help with vital signs stations.. it is not part of my job but I do it anyway .. I refer my patients and the nurses see them .. So there is teamwork .. The support system is great so I never felt pressure to leave my wBOT duties<br>**FDG**<br>"And sometimes when you go with the OTL, the patients are feeling uncomfortable because she shouts at them. And we are unable to tell her that what you are doing is wrong, you are not supposed to talk like that with patients. Now the patients are telling us that we are not welcomed in their houses if we come with the OTL."
Level of knowledge of CHWs	Depends on education of the individual - some will do as you ask, others you need to dig deeper ..<br>they are done some of the training (phase 1 and 2)<br>Are they able to implement it ? some are .. others are dragging, but they are trying p7<br>**FDG – what cases aren't you able to deal with?**<br>Sick patient with no food >refer<br>Elderly patients who needs to be in old age home
Travel (time, mode, particular features/challenges)	
Resources Available	
Resources Needed	Have to buy batteries, transport, airtime (although do call-backs), forms, uniform out of own money (although towards the end, the CHW were wearing pink shirts)<br>Don't have office space, or masks for working with TB patients<br>forms .. yes, one day a teacher was here from the primary school.. and I asked him to make copies<br>*No photocopier*<br>They need a permanent structure so that they can do maternity<br>We are using our own transport - that is what is irritating<br>Shortage of bags

For peer review only

	I am suing my own petrol, my own cellphone, chw use their own airtime.. sometimes they even call patients.<br>We need a place to meet as CHW /OTLS; patient files are stored in PN's room<br>We need our own social worker<br>**FDG – description of the bag**<br>We are meant to screen everybody over 25 years for diabetes.. but we don't because the strips are not enough<br>The machine is already broken .. it is not strong<br>We don't have an office, airtime, batteries (we have to buy out of our money)<br>**FDG:** we were given 10 bags for 17 of us; my machine isn't working.. I reported it .. it is embarrassing when it doesn't work; the strips are finished; we report to the sister who says that the patient must come to the clinic<br>You use your own airtime to call an ambulance;<br>If you are tracing, if you don't find that person at the address, you call them using your own airtime .. otherwise how are going to find them What makes us not to achieve more is that when we come to the clinic and ask for some more equipment, we are told that that they don't have'
Community relationships	Initially not accepted, were chased away .. now they are accepted. ..have built rapport<br>community unhappy when CHW on strike (they were saying nurses were on strike p6<br>can do dressings which patients appreciate<br>• The relationship between the community and the CHW .. 'one day we were out walking with Dr V and Dr O from western Africa.. and somebody asked the CHW how come you are walking with so many men' .. she answered they are doctors we are working together ' Dr O asked the person .. how do you know my nurse? .. she brings me medication every month P6 ..Dr O was very excited that they were well known in the community p6<br>• That is why we allocate houses to them in their area.. because then people can call them over the weekend . and then they give us a report and say .. a patient was giving birth .. and so we called an ambulance etc .. it is better if the CHW is in the area<br>• We don't have a clinic committee .. but we have a very good relationship with the ward councilor (Area 1?) (EN02 as well – if there is somebody who is sick he calls me, so we work together)<br>• I want to start a support group for young people with HIV<br>The community is aware of us .. they are coming to us. Even the referral forms its good<br>People are taking treatment now. I think there is lesser tracing on ARV patients, most defaulters you will find are TB. Our stats are going up<br>**Reason for success**<br>Employing us as staff nurses .. cos if the CHW see you as supporting them, trying to understand where they are coming from, especially with this employer thing is mixed up … That is why they refused to go on strike today<br><br>When they were on strike for 3 months .. the patients were complaining .. where have you been, we have been waiting for you .. they decided not to go to the clinic but wait until the sisters were back .. so they really rely on them<br>**FGD**<br>We get the list of defaulters .. but now there are few because of our work .. people have now accepted their illness .. people do appreciate us.. just come and disclose to us .. and later that person will come and thank you for the work you have done p5<br><br>Iyhoo, I'm so proud about my patients, they get better. Others I found them bedridden but because of us they have recovered. Even if they don't give me any material thing, but when I pass them on the street and she/he tells others that she/he is walking because of me, I feel satisfied because at least I have made other people better." P15<br><br>"In Area 1 we don't have any conflict with the community because the ward councilor had introduced us during the public meeting. They know us and they trust us in a way that when they need help even if it is during the night, they wake us. People got that belief that when the CHWs call the ambulance, the ambulance arrives quickly. So we are don't mind because we know the community rely on us."<br><br>We already said, they are able to come to us. Like no the nurse at the clinic is unapproachable, she talks to the patients the way she likes and the patient keep quiet but when they see us, they tell us what happened with the nurse at the clinic. So it is easy for them to talk to us than to the nurses. Other patients said if the nurse comes to their houses and speaks the way she speaks when she is at the clinic, we are going to hit her. Then we try to calm them down." P15

For peer review only

	If my neighbour is sick I ask my partner to attend to her.. cos I don't want the neighbour to say that I talk about her ..<br><br>"The community things we are their helper, they love us. In everything that we do, they stand with us." P25<br><br>"To honest the patients trust us and they appreciate us. I have a patient who is HIV and she doesn't want to come to the clinic and I don't want my patient to default. So I promised her that I will assist. Then I came to the clinic and talk to the sister. I said sister my patient is working at the farms so please pack medication for her, here is her clinic card. I'll ask her to come next month. The sister scolded me because the patient did not come to the clinic on her last appointment. So I begged the sister and I promised the sister that I will bring the patient myself. Then it was sorted, the patient came to the clinic with me and she got her medication." P18
Relationships with other NGOs, networking, knowledge of context (draw on what exists & links clients)	We work with policing forum, ward counsellor, we have their numbers .. we do campaigns together .. community and health campaigns (e.g.creches)<br>Household registration for municipality .. although you ask these questions about income.. they think you are bringing food .. so they make you feel like you are making empty promises)<br>AGAPe – I have referred too many people there .. but there results are not good.. if people don't have an ID they come back with nothing. We need our own social worker
Overall impression<br>- Incl do CHWs feel valued?<br>- Other notes	During the strike some of the CHW tried to work .. but the EN and the FM told them not to<br>strike started again today but CHW refuse to be part of strike (strike is for better employment issues, high bank card deductions (20R), lack of communication from smart purse, uniform); eventually they had to accepted it .. so not joining strike today<br><br>**Can the programme be led by staff nurses ?**<br>I am saying this because with staff nurses in the community … I can deal with almost 90% of the problems .. but as a staff nurse I am not allowed to give out treatment.. if I pre-pack medication .. it has to be checked by the professional nurse .. also I can't consult
Interesting points	• FM is involved, and knows how the WBOT programme runs, clinic was set up and the same time as the WBOT<br>• EN02 know there is a reciprocal relationship between WBOT and clinic and need to maintain that ..(helps when clinic is busy and they see her patients)<br>• If clinic is not welcoming to patients .. difficulty in persuading patients to go to the clinic .. So success of WBOT is dependent on quality of care in clinics<br>• In EN only model – a PN has to be available to dispense the medication (or check it?)<br>• It seems that EN02 who goes out into the community has a better relationship with the CHW, the trust each other more .. a lot of the problems are about lack of trust<br>• EN 01 is a lot more passive and prefers to work in the clinic;<br>• PN sees patients but doesn't seem to meet with CHW when they return .. EN02 knows households and really works with CHW<br><br>How does X model compare with other sites .. as they are not doing medication delivery<br>Affects access, relationship with community, machines etc, number of households you can do, actual activities that they do

Standards for Reporting Qualitative Research checklist

No	Topic	Item	Whether included in the manuscript
S1	Title	Concise description of the nature and topic of the study Identifying the study as qualitative or indicating the approach (e.g., ethnography, grounded theory) or data collection methods (e.g., interview, focus group) is recommended	Yes
S2	Abstract	Summary of key elements of the study using the abstract format of the intended publication; typically includes background, purpose, methods, results, and conclusions	Yes
**Introduction**			
S3	Problem formulation	Description and significance of the problem/phenomenon studied; review of relevant theory and empirical work; problem statement	Yes
S4	Purpose or research question	Purpose of the study and specific objectives or questions	yes
**Methods**			
S5	Qualitative approach and research paradigm	Qualitative approach (e.g., ethnography, grounded theory, case study, phenomenology, narrative research) and guiding theory if appropriate; identifying the research paradigm (e.g., postpositivist, constructivist/ interpretivist) is also recommended; rationale	Yes
S6	Researcher characteristics and reflexivity	Researchers' characteristics that may influence the research, including personal attributes, qualifications/experience, relationship with participants, assumptions, and/or presuppositions; potential or actual interaction between researchers' characteristics and the research questions, approach, methods, results, and/or transferability	Yes; Research characteristics are listed at the end of the article
S7	Context	Setting/site and salient contextual factors; rationale	Yes
S8	Sampling strategy	How and why research participants, documents, or events were selected; criteria for deciding when no further sampling was necessary (e.g., sampling saturation); rationale	Yes
S9	Ethical issues pertaining to human subjects	Documentation of approval by an appropriate ethics review board and participant consent, or explanation for lack thereof; other confidentiality and data security issues	Yes
S10	Data collection methods	Types of data collected; details of data collection procedures including (as appropriate) start and stop dates of data collection and analysis, iterative process, triangulation of sources/methods, and modification of procedures in response to evolving study findings; rationale	Yes
S11	Data collection instruments and technologies	Description of instruments (e.g., interview guides, questionnaires) and devices (e.g., audio recorders) used for data collection; if/how the instrument(s) changed over the course of the study	Yes
S12	Units of study	Number and relevant characteristics of participants, documents, or events included in the study; level of participation (could be reported in results)	Yes
S13	Data processing	Methods for processing data prior to and during analysis, including transcription, data entry, data management and security, verification of data integrity, data coding, and anonymization/deidentification of excerpts	Yes
S14	Data analysis	Process by which inferences, themes, etc., were identified and developed, including the researchers involved in data analysis; usually references a specific paradigm or approach; rationale	Yes
S15	Techniques to enhance trustworthiness	Techniques to enhance trustworthiness and credibility of data analysis (e.g., member checking, audit trail, triangulation); rationale	Yes
**Results**			
S16	Synthesis and interpretations	Main findings (e.g., interpretations, inferences, and themes); might include development of a theory or model, or integration with prior research or theory	Yes
S17	Links to empirical data	Evidence (e.g., quotes, field notes, text excerpts, photographs) to substantiate analytic findings	Yes
**Discussion**			
S18	Integration with prior work, implications, transferability and contribution to the field	Short summary of main findings; explanation of how findings and conclusions connect to, support, elaborate on, or challenge conclusions of earlier scholarship; discussion of scope of application/ generalizability; identification of unique contribution(s) to scholarship in a discipline or field	Yes
S19	Limitations	Trustworthiness and limitations of findings	Yes
**Other**			
S20	Conflicts of interest	Potential sources of influence or perceived influence on study conduct and conclusions; how these were managed	Yes

For peer review only

S21	Funding	Sources of funding and other support; role of funders in data collection, interpretation, and reporting	Yes

For peer review only
